# Microbial-Host Co-metabolites Are Prodromal Markers Predicting Phenotypic Heterogeneity in Behavior, Obesity, and Impaired Glucose Tolerance

**DOI:** 10.1016/j.celrep.2017.06.039

**Published:** 2017-07-05

**Authors:** Marc-Emmanuel Dumas, Alice R. Rothwell, Lesley Hoyles, Thomas Aranias, Julien Chilloux, Sophie Calderari, Elisa M. Noll, Noémie Péan, Claire L. Boulangé, Christine Blancher, Richard H. Barton, Quan Gu, Jane F. Fearnside, Chloé Deshayes, Christophe Hue, James Scott, Jeremy K. Nicholson, Dominique Gauguier

**Affiliations:** 1 Division of Computational and Systems Medicine, Department of Surgery and Cancer, Faculty of Medicine, Imperial College London, Sir Alexander Fleming Building, Exhibition Road, South Kensington, London SW7 2AZ, UK; 2 Wellcome Trust Centre for Human Genetics, University of Oxford, Roosevelt Drive, Oxford OX3 7BN, UK; 3 Cordeliers Research Centre, INSERM UMR_S 1138, University Pierre & Marie Curie and University Paris Descartes, Sorbonne Paris Cité, Sorbonne Universities, 15 Rue de l’École de Médecine, 75006 Paris, France; 4 Department of Medicine, Imperial College London, Du Cane Road, London W12 0NN, UK

**Keywords:** natural phenotypic variation, impaired glucose tolerance, obesity, anxiety, microbiome, metabolome, transcriptome, trimethylamine-*N*-oxide, TMAO, endoplasmic reticulum stress, insulin secretion

## Abstract

The influence of the gut microbiome on metabolic and behavioral traits is widely accepted, though the microbiome-derived metabolites involved remain unclear. We carried out untargeted urine ^1^H-NMR spectroscopy-based metabolic phenotyping in an isogenic C57BL/6J mouse population (n = 50) and show that microbial-host co-metabolites are prodromal (i.e., early) markers predicting future divergence in metabolic (obesity and glucose homeostasis) and behavioral (anxiety and activity) outcomes with 94%–100% accuracy. Some of these metabolites also modulate disease phenotypes, best illustrated by trimethylamine-*N*-oxide (TMAO), a product of microbial-host co-metabolism predicting future obesity, impaired glucose tolerance (IGT), and behavior while reducing endoplasmic reticulum stress and lipogenesis in 3T3-L1 adipocytes. Chronic in vivo TMAO treatment limits IGT in HFD-fed mice and isolated pancreatic islets by increasing insulin secretion. We highlight the prodromal potential of microbial metabolites to predict disease outcomes and their potential in shaping mammalian phenotypic heterogeneity.

## Highlights

• High-fat diet drives phenotypic heterogeneity in metabolism and behavior
• Microbial metabolites, including methylamines, predict phenotypic heterogeneity
• TMAO attenuates ER stress and reduces lipogenesis in adipocytes
• TMAO improves insulin secretion and restores glucose tolerance in vivo

## Graphical Abstract

**Figure:**
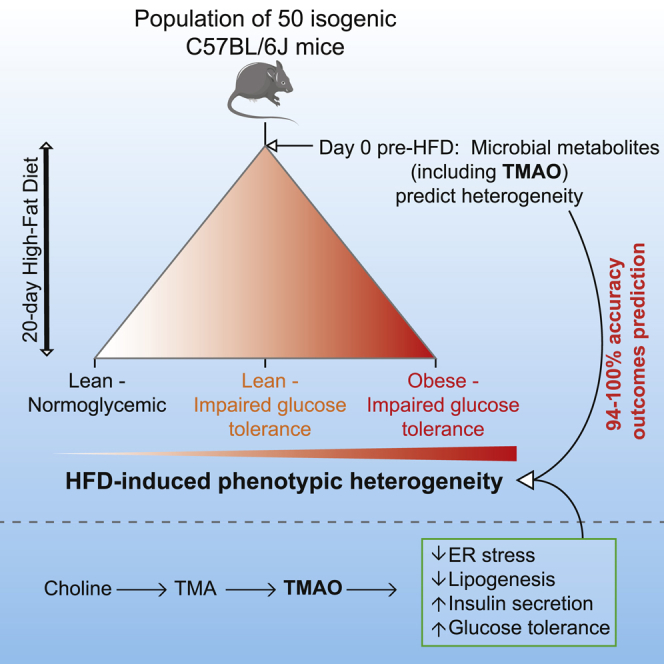

## Teaser

Dumas et al. study the metabolic and behavioral phenotypic heterogeneity induced by a high-fat diet intervention in an isogenic mouse population model. Using ^1^H-NMR spectroscopy, they identify pre-interventional urinary metabolic signatures (including microbial-host co-metabolites) predicting future phenotypic heterogeneity. In particular, TMAO corrects endoplasmic reticulum stress and glucose tolerance.

## Introduction

Phenotypic heterogeneity is generally attributed to gene-environment interactions. However, phenotype variability is also commonly observed in identical twins and in isogenic model systems (Lehner, 2013), which can be exacerbated by high-fat diet (HFD) feeding in mice (Burcelin et al., 2002). This phenomenon is associated with changes in gut microbial communities in isogenic mouse populations (Serino et al., 2012) and in monozygotic twins (Ridaura et al., 2013). With ∼10 million genes (Li et al., 2014), there is growing evidence that the gut microbiome contributes to obesity (Cotillard et al., 2013, Le Chatelier et al., 2013, Turnbaugh et al., 2006) and type 2 diabetes (Karlsson et al., 2013, Qin et al., 2012) in the context of Western-style diets rich in saturated fats (David et al., 2014, Muegge et al., 2011). Fecal microbiota transplantations (Smith et al., 2013, Turnbaugh et al., 2006) and metagenomic studies have highlighted the roles of microbiome architecture and richness (Cotillard et al., 2013, Le Chatelier et al., 2013).

However, beyond beneficial bacteria (Dao et al., 2016, Shoaie et al., 2015), the microbiome-derived mediators promoting host health or disease remain elusive: a few microbial metabolite families (e.g., short-chain fatty acids or bile acids) are known to affect human health (Dumas et al., 2014, Russell et al., 2013). To drive a shift in host physiology and potentially affect pathogenesis, microbial metabolite variation should precede changes in host metabolism and physiology and these metabolites should directly modulate traits associated with the disease. In this context, phenotypic heterogeneity observed in discordant twins or in populations of isogenic mice fed HFD offers a unique opportunity to evaluate microbial metabolites as early predictive (i.e., prodromal) markers of disease onset and progression and to assess their impact on disease (Hsiao et al., 2013, Venkatesh et al., 2014, Yoshimoto et al., 2013).

To evaluate microbial metabolites as prodromal markers, we repurposed a pharmaco-metabonomics framework (Clayton et al., 2006), which we developed initially for drug toxicity prediction using pre-dose metabolic phenotypes, to predict complex metabolic and behavior phenotype outcomes following HFD feeding in isogenic mouse populations. We best exemplify the influence of microbial-host co-metabolites through trimethylamine-*N*-oxide (TMAO), a phase 1 oxidation product of gut microbial trimethylamine (TMA) that we observed first in insulin resistance (Dumas et al., 2006) and then with *Akkermansia muciniphila*’s beneficial effects on impaired glucose tolerance (Plovier et al., 2017) and that plays roles in atherosclerosis (Koeth et al., 2013, Tang et al., 2013, Wang et al., 2011b). In our study, methylamines predict impaired glucose tolerance (IGT) and obesity outcomes. TMAO reduces endoplasmic reticulum (ER) stress and lipogenesis in adipocytes, increases insulin secretion in isolated pancreatic islets, and attenuates diet-induced IGT, thus demonstrating dual prodromal and functional properties of microbiome-derived metabolites in health and disease (Dumas, 2011, Nicholson et al., 2012).

## Results

### Phenotypic Heterogeneity Underpins IGT and Obesity in Isogenic Mouse Populations

To study the phenomenon of heterogeneous metabolic adaptation to HFD in mice (Burcelin et al., 2002, Serino et al., 2012), we generated a large population of isogenic C57BL/6J mice fed either chow diet (CHD) or HFD (n = 193) for up to 5 months. HFD-fed mice became divergent from CHD-fed mice for IGT assessed by intraperitoneal glucose tolerance tests (IP-GTTs) and body weight phenotypes with strong and permanent heterogeneity in glucose tolerance and body weight (Figures S1A–S1C). Phenotype SDs for body weight (BW) and IGT progressively increased between 3 and 5 months of fat feeding and were greater in HFD-fed mice than in CHD-fed mice (Figure S1D). These preliminary results show that phenotypic heterogeneity develops progressively over time and confirm that HFD feeding promotes this phenomenon.

**Figure 1:**
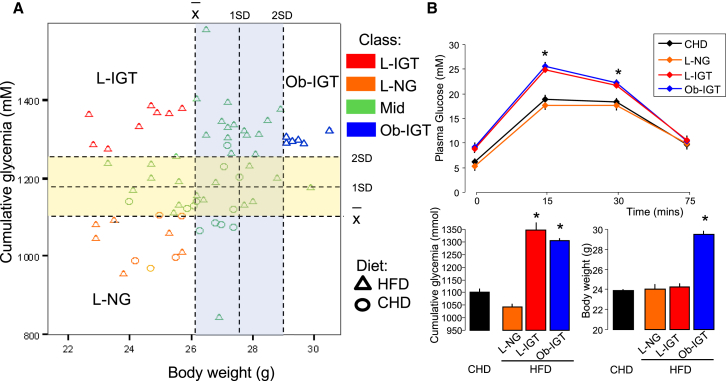
HFD Induces Phenotypic Heterogeneity in an Isogenic Population of 50 C57BL/6J Mice within 3 Weeks (A) HFD feeding caused segregation of physiological phenotypes for diabetes (cumulative glycemia) and obesity (BW), and thresholds on cumulative glycemia and BW stratify the population into three disease sub-phenotypes: lean normoglycemic (*LNG*), lean impaired glucose tolerance (*L-IGT*), and obese impaired glucose tolerance (*Ob-IGT*). (B) Short-term (3 weeks) HFD feeding generates heterogeneous phenotypes for BW and glucose tolerance in C57BL/6J male mice. Age-matched controls were fed a standard carbohydrate diet (CHD, n = 85–95). Data are presented as means ± SE. ^∗^p < 0.05, ^∗∗^p < 0.01, ^∗∗∗^p < 0.001. See also Figures S1–S3.

We then bred a new cohort of 50 isogenic mice for in-depth characterization of the dietary-induced phenotype heterogeneity from 3 weeks of HFD feeding onward (Figure S1D). Outcomes from IP-GTT performed after 3 weeks of HFD feeding (Figure 1; Figure S2) and BW (Figure 1; Figure S3) were used to stratify the mouse population according to glucose tolerance (cumulative glycemia during the IP-GTT) and obesity phenotypes. Applying a threshold of 2 SD above the mean of cumulative glycemia and BW defined three disease sub-groups of extreme responders to HFD feeding: lean with impaired glucose tolerance (*L-IGT*), obese with impaired glucose tolerance (*Ob-IGT*), and non-responder lean normoglycemic (*LNG*) (Figure 1A). As expected, fasting glycemia in *LNG* mice (5.22 ± 0.35 mM) was not different from that of CHD-fed controls (6.14 ± 0.11 mM). Identical glycemic profiles during the IP-GTT in fat-fed *LNG* mice and in control CHD-fed mice confirms the resistance of *LNG* mice to the dietary challenge (Figure 1B). In addition, fasting glycemia was significantly lower in these groups than in *L-IGT* mice (9.12 ± 0.87 mM, p = 0.001) and *Ob-IGT* mice (8.90 ± 0.67 mM, p < 0.001) (Figure 1B; Figure S2). Fat-fed mice from the *Ob-IGT*, *LNG*, and *L-IGT* groups were identified in parallel in several cages, thus ruling out possible cage effects (Ridaura et al., 2013) on phenotypes.

### Extreme HFD Responders Have Altered Insulin Secretion, Adiposity, and Lipids

To extend the in vivo physiological screening of extreme responders, we determined insulin during the IP-GTT, adiposity index (ratio of adipose weight to body weight), and plasma lipids. Even though glucose-stimulated insulin secretion was not used for stratification of the mouse groups, the glucose-intolerant groups *L-IGT* and *Ob-IGT* are hyperinsulinemic compared to *LNG* mice (Figures S2B and S2E). Pre-intervention BWs at 5 weeks were not significantly different among any of these sub-groups, but BMI, BW gain, and the weights of the epididymal fat pad (EPD), retroperitoneal fat pad (RFP), and brown adipose tissue (BAT) diverged in the *Ob-IGT* group compared to other groups (p < 0.001) (Figures S3A–S3I) at day 20. The obese group had significantly lower plasma high-density lipoprotein (HDL) and higher plasma triglycerides than the lean groups, while the glucose-intolerant group *L-IGT* had significantly more plasma low-density lipoprotein (LDL) than the *LNG* mice, suggestive of stratification-associated dyslipidemia in obese (Ob) and IGT mice (Figures S3J–S3M).

### HFD Induces Heterogeneity in Behavior

Because mice exhibiting extreme glucose tolerance and body weight were systematically observed in different cages, we hypothesized that heterogeneous metabolic adaptation to HFD may involve behavioral traits, which we characterized using robust procedures in *Ob-IGT*, *LNG*, and *L-IGT* mice (Figure S4). Time spent in the elevated plus maze (EPM) closed arms and latency to enter the open field (OF) central arena show similar patterns, supporting inter-test validity. EPM activity and anxiety generally increased with IGT between *LNG* and *Ob-IGT* mice (Figures S4A and S4B). This was reflected by the increased number of entries in the closed EPM arms in *Ob-IGT* mice (11.7 ± 1.0) compared with *LNG* mice (8.8 ± 0.8) (p = 0.026) and the increased time spent in the EPM center in *Ob-IGT* mice (76.5 ± 8.7) and *L-IGT* mice (80.5 ± 4.8) compared with *LNG* mice (59.0 ± 7.3) (p < 0.05). *Ob-IGT* mice also showed a significantly higher number of rearings (p = 0.04) and transitions in the OF when compared to lean mice. Activity parameters in the OF (number of rearings and transition) were increased in *Ob-IGT* mice compared to *LNG* mice (p = 0.04) (Figures S4C and S4D). Altogether, these results show that HFD induces heterogeneous metabolic, hormonal, and behavioral changes characterized by increased anxiety and activity in mice showing impaired glucose homeostasis and increased BW.

### Metabolic Phenotypes Mirror Phenotypic Variability

**Figure 2:**
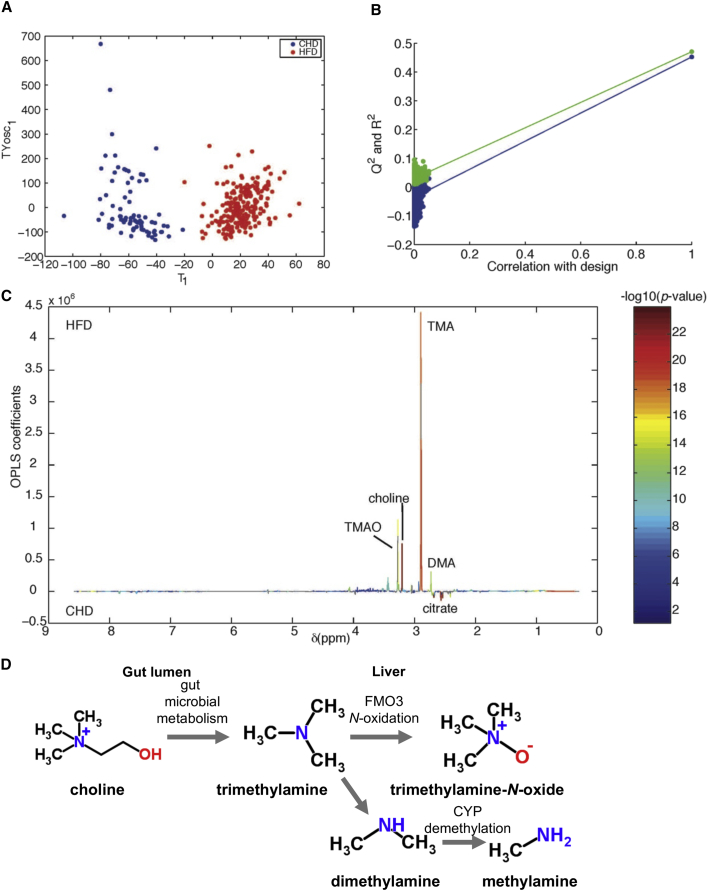
The Urinary Metabolic Signature of HFD in the C57 Mouse (A) O-PLS-DA scores plot. (B) O-PLS-DA permutation plot. The O-PLS-DA model was validated by random permutations (n = 10,000 iterations) of the original variable to explain class membership (CHD versus HFD). The horizontal axis represents the correlation between the original class membership (right) and the randomly permuted class membership vectors (no longer correlated with the original class membership) (left). The y axis represents the goodness-of-fit R^2^ (in green) parameter obtained for each O-PLS-DA model and the goodness-of-prediction Q^2^ (in blue) parameter obtained by 7-fold cross-validation of the O-PLS-DA model. The R^2^ and Q^2^ parameters for the original model in the top right corner do not belong to the population of 10,000 models fitted with random class memberships, highlighting that the original model does not belong to the population of 10,000 randomly permuted models (p < 0.0001) and thereby confirming the significance of the fitness and prediction ability attached with the original O-PLS-DA model. (C) O-PLS-DA model coefficient plot. (D) Summary of microbial-mammalian co-metabolism of methylamines.

To identify metabolic signatures associated with heterogeneous adaptation to HFD, we performed ^1^H-NMR-based untargeted metabolic phenotyping (i.e., metabotyping) (Gavaghan et al., 2000) of 24 hr urinary collections obtained at baseline before dietary intervention (5 weeks of age, day 0), and 1, 2, and 20 days (8 weeks of age) after intervention. An orthogonal partial least-squares discriminant analysis (O-PLS-DA) constructed using all urines clearly discriminated CHD from HFD samples (p = 10^−4^) (Figure 2A). The O-PLS-DA model was highly predictive when randomly resampled 10,000 times (Figure 2B), and detailed structural assignment (Table S1) confirmed that the methylamine pathway is activated in HFD (Figure 2C), as initially reported (Dumas et al., 2006): TMA is derived from dietary choline fermentation by commensal bacteria, and metabolized into TMAO, dimethylamine (DMA), and monomethylamine (MMA) in the liver (Figure 2D) (al-Waiz et al., 1992, Craciun and Balskus, 2012, Dolphin et al., 1997).

### Predictive Modeling of Disease Sub-groups and Quantitative Phenotypes

**Figure 3:**
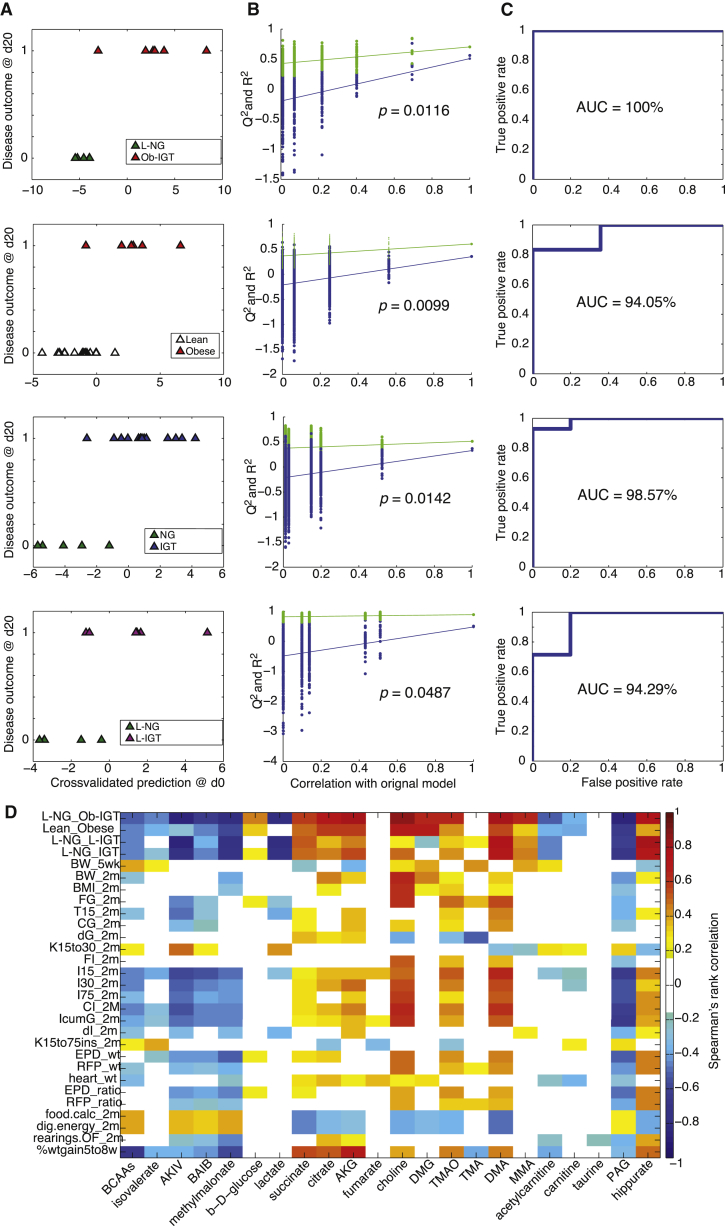
Pre-intervention Metabotypes Predict Disease Outcome and Phenotypic Heterogeneity (A) Pre-intervention (day 0) urinary ^1^H-NMR-based metabolic profiles predict disease outcome (day 20, after a 3-week HFD challenge). (B) Model goodness-of-fit (R^2^) and goodness-of-prediction (Q^2^) parameters are significantly different from those expected by chance in a permutation test (empirical p value derived from 10,000 iterations). (C) Receiver-operating characteristic (ROC) curves demonstrate efficient prediction of future disease outcomes. (D) Heatmap of significant metabolic predictors of disease and quantitative phenotype outcomes present complex yet structured patterns (see Table S1 for assignments). The heatmap was built using significant Spearman’s rank-based correlations after 10,000 random permutation testing between day 0 urinary metabolites and day 20 heterogeneous phenotypes. Only significant correlations (permutation testing p < 0.05) are color-coded on the heatmap; non-significant correlations are left uncolored (white). BCAA, branched chain amino acids. Phenotypes were determined in mice at 5 weeks (5wk) or 2 months (2m). Glucose tolerance tests were used to determine glycemia 15 min after glucose injection (T15); cumulative glycemia (CG); cumulative glycemia above baseline (dG); the disappearance rate of glucose from blood, in minutes (K15to30 and K15to75ins); insulin secretion 15, 30, and 75 min after glucose injection (I15, I30, and I75, respectively); cumulative insulinemia (CI); cumulative insulinemia above baseline (dI); and the ratio of cumulative glycemia to cumulative insulinemia (IcumG). BW, body weight; BMI, body mass index; FG, fasting glycemia; FI, fasting insulinemia; AUC, area under the curve during the intraperitoneal glucose tolerance test (IP-GTT); EPD, epididymal fat pad; EDP_ratio, EPD weight to BW ratio; RFP, retroperitoneal fat pad; RFP_ratio, RFP weight to BW ratio; L, Lean; Ob, obese; IGT; impaired glucose tolerance; OF, open field; dig.energy, digestible energy; food.calc, food intake; AKIV, alpha-keto-isovalerate; BAIB, beta-aminoisobutyrate; AKG, 2-oxoglutarate; DMG, *N*-*N*-dimethylglycine. See Table 1 for other abbreviations.

To test whether pre-intervention metabotypes can predict future disease outcome, we built a series of O-PLS-DA models predicting disease sub-groups after 20-day HFD from baseline urinary metabolic phenotypes at day 0. We implemented a 7-fold cross-validation strategy to assess the performance of the models: the cross-validated score plots show a clear prediction of glycemia and obesity sub-phenotypes (Figure 3A). We also resampled our predictions using 10,000 random permutations and rederived goodness-of-prediction Q^2^_Yhat_ parameters by 7-fold cross-validation, demonstrating the original O-PLS-DA models were significantly different from 10,000 random cross-validated models, with p = 0.0099–0.0487 (Figure 3B). We then evaluated the performance of the predictive O-PLS-DA cross-validated scores in a receiver-operating characteristic (ROC) analysis. The area under the ROC curve (AUC), corresponding to discriminative power ranged from 94% to 100% (Figure 3C). We built and permutation validated a series of predictive O-PLS-DA models showing significant segregation of the original models for the following sub-phenotypes: extreme (Q^2^_Yhat_ = 0.67, p = 0.0116, AUC = 100%), lean from Ob (Q^2^_Yhat_ = 0.36, p = 0.0099, AUC = 94%), normoglycemic from IGT (Q^2^_Yhat_ = 0.37, p = 0.0142, AUC = 98.6%), and *LNG* from *L-IGT* (Q^2^_Yhat_ = 0.50, p = 0.0487, AUC = 94.29%).

We also computed O-PLS regressions between urinary metabotypes and the 44 physiological or behavioral quantitative phenotypes measured at day 20 using the whole cohort of HFD-fed mice (Table 1). Permutation testing showed that 25 of 44 quantitative phenotypes after 3 weeks of HFD feeding were significantly predicted using baseline (i.e., before HFD challenge) urinary metabotypes. In particular, pre-interventional metabotypes predict not only BW at baseline (p = 0.0007) and after a 3-week HFD (p = 0.0034) but also BMI (p = 0.0362), BW gain (p = 0.0001), fasting glycemia (p = 0.012), cumulative glycemia and insulinemia, heart and fat pad (EPD and RFP) weights, and even behavioral traits (number of rearings in OF, p = 0.0122). These data suggest that HFD heavily disturbs microbial and host metabolism overnight with gradual and often phenotype-specific heterogeneity.

**Table 1:** Predictions for Quantitative Physiological and Disease Phenotypes

Phenotype Day 20	Day 0 (p Value)	Day 1 (p Value)	Day 2 (p Value)	Day 20 (p Value)
BW baseline (day 0)	0.0007	0.0001	0.0001	NS
BW	0.0034	0.0005	0.0012	0.0269
BMI	0.0362	0.0054	0.0005	0.0059
Fasting glycemia	0.0012	0.0079	NS	0.0121
Glycemia 15 min IP-GTT	0.0008	0.0429	0.0341	0.0105
Glycemia 30 min IP-GTT	NS	NS	NS	0.0269
Glycemia 75 min IP-GTT	NS	0.0174	NS	NS
AUC glycemia IP-GTT	0.0246	NS	NS	0.0282
Delta glycemia IP-GTT	0.0133	0.0109	NS	NS
K parameter (glycemia) IP-GTT	0.0236	0.0225	0.0055	NS
Fasting insulinemia	0.0095	NS	0.041	NS
Insulinemia 15 min IP-GTT	0.0001	0.0004	0.0019	0.0028
Insulinemia 30 min IP-GTT	0.0009	NS	0.0028	NS
Insulinemia 75 min IP-GTT	0.0012	NS	NS	0.033
AUC insulinemia IP-GTT	0.0001	0.0411	0.0051	0.0266
AUC(I)/AUC(G) IP-GTT	0.0002	NS	0.0113	NS
Delta insulinemia IP-GTT	0.0154	0.0034	0.0012	NS
K parameter (insulin) IP-GTT	0.0122	0.0411	0.0125	0.0152
EPD weight	0.0146	NS	0.0057	0.014
RFP weight	0.0243	NS	NS	NS
BAT weight	NS	NS	0.0368	0.0024
Heart weight	0.0001	0.0005	0.0109	0.0392
EPD weight/BW (%)	0.0058	NS	0.0024	0.0102
RFP weight/BW (%)	0.0343	NS	NS	0.0341
BAT weight/BW (%)	NS	NS	NS	0.0034
Heart weight/BW (%)	NS	NS	NS	0.0175
Food intake	0.0051	0.0228	0.0008	NS
Entries open arm	NS	NS	NS	0.0295
Time to enter open arm	NS	0.0388	NS	0.0155
Digestible energy	0.0042	0.0233	0.0008	NS
Rearings (OF)	0.0122	0.0374	0.0489	NS
BW gain day 0–20	0.0001	0.0001	0.0001	NS

O-PLS regression models predicting each quantitative phenotype variable using metabolic profiles (n = 44) at a given time point (day 0, day 1, day 2, or day 20) were assessed by permutation testing. The p values for the Q^2^_Yhat_ model prediction parameter were obtained by random permutation testing with 10,000 iterations. Then, the original Q^2^_Yhat_ is projected on the confidence interval of the population of 10,000 Q^2^_Yhat_ values obtained from each random model to derive a non-parametric empirical p value. BW, body weight; AUC, area under the curve during the intraperitoneal glucose tolerance test (IP-GTT); EPD, epididymal fat pad; RFP, retroperitoneal fat pad; BAT, brown adipose tissue; OF, open field; NS, not statistically significant.

### Prodromal Predictors of Disease Outcomes and Quantitative Traits

We next identified metabolic markers for phenotypic heterogeneity using empirical p values generated for Spearman’s rank correlation with 10,000 random permutations. The patterns of association between urinary metabolites and physiological phenotypes are complex, with multiple partial contributions from one metabolite to each phenotype (Figure 3D). For instance, heart weight is predicted by increased excretion of tricarboxylic acid (TCA) cycle intermediates (citrate, 2-oxoglutarate, fumarate, and succinate), as well as choline and *N*-*N*-dimethylglycine (DMG). We show that excretion of gut microbial metabolites, including methylamines, predicts several physiological and behavioral traits (Figure 3D). Pre-interventional excretion of choline, TMAO, DMA, and MMA predicts not only IGT and obesity but also stratification of the mouse population into *Ob-IGT* and *L-IGT* disease sub-groups and finally heterogeneity of quantitative phenotypes, i.e., fat pad (EPD and RFP) weights (raw and normalized to total BW), BMI, or food consumption traits. Baseline TMAO excretion predicts obesity and IGT outcomes (BW, BMI, BW gain, EPD weight and ratio, glycemia, and insulinemia). In contrast, urinary TMA presents weaker associations and is negatively correlated to disease sub-groups (Figure 3D). Methylamines (TMA, DMA, MMA, and TMAO) and their pre-cursor choline are the major metabolites associated with HFD in the C57BL/6J mouse (Figure 2D), which is consistent with our previous report (Dumas et al., 2006). Other microbial-host co-metabolites, such as phenylacetylglycine (PAG) and hippurate, are also predictive of disease risk, BW, glycemia, insulinemia, feeding behavior, and anxiety parameters, suggesting that symbiotic metabolism also predicts future anxiety and activity patterns (Figure 3D).

### TMAO Correlates with Lower Adipose ER-Stress Response and Insulin Signaling

**Figure 4:**
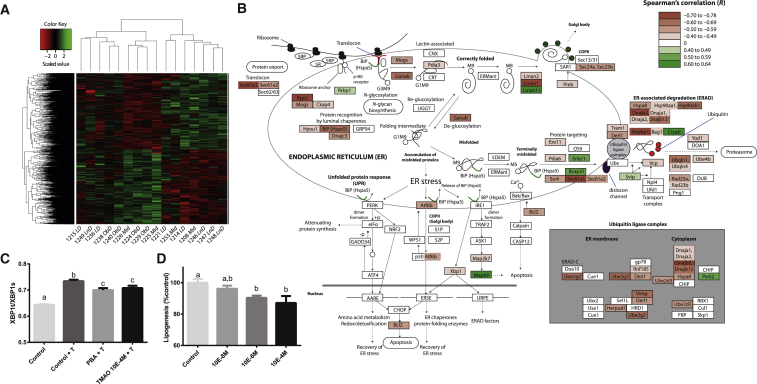
TMAO Alleviates ER Stress (A) The 2,875 EPD genes significantly correlate with TMAO excretion after 20-day HFD. See also Table S2. (B) TMAO correlates with reduced expression of ER stress genes. See also Tables S3 and S4. (C) TMAO reduces ER stress in differentiating 3T3-L1 adipocytes. Data were derived from six replicates of three cell preparations. (D) TMAO reduces lipid accumulation in 3T3-L1 adipocytes. Data were derived from six replicates of three independent cell preparations. Data are presented as means ± SEM. T, tunicamycin; PBA, 4-phenylbutyrate. A one-way ANOVA was performed to identify significantly different factor levels, denoted as a–c (p < 0.05 post hoc test). The pathway of protein degradation in the ER was redrawn from Kyoto Encyclopedia of Genes and Genomes (KEGG), with KEGG annotations substituted for gene annotations in cases of significant differences.

We focused on TMAO as the product of the main microbial-host co-metabolic pathway significantly associated in our study (choline, TMA, DMA, MMA, and TMAO) (Figures 2 and 3). Details of TMAO action on obesity and adipocyte function remain unknown. After 3 weeks of HFD, TMAO excretion is negatively associated with EPD weight and the ratio of EPD weight to body weight observed 20 days post-HFD (r = −0.302 and −0.291, respectively). We profiled the transcriptome in EPD from HFD-fed mice belonging to the *LNG*, *LD*, and *Ob-IGT* continuum and characterized by glucose tolerance and body weight within 2 SD of the mean for these phenotypes (i.e., midgroup) (Figure 4; Table S2). Permutation testing identified 2,875 genes correlated with TMAO excretion at day 20 (Figure 4A; Table S2). This transcriptomic correlation pattern accounts for combined effects of TMAO and other metabolites associated with extreme adaptations of mice to HFD (e.g., hippurate and PAG) and consequences of altered physiological and behavioral phenotypes. A gene ontology analysis (Table S3) highlights a coordinated regulation involving response to endoplasmic reticulum stress, regulation of lipid biosynthetic process, insulin receptor signaling pathway, and fat cell differentiation. Protein processing, ER-associated degradation, and ubiquitin-ligase complex are strikingly anti-correlated with TMAO (Figure 4B; Table S3). Under ER stress conditions, *Ire1* cleaves a 26-nucleotide intron from the *Xbp1* mRNA (*Nfx1*, r = −0.51, p = 0.0456), leading to spliced *Xbp1* mRNA encoding for a transcription factor promoting expression of unfolded protein response (UPR) genes (Hotamisligil, 2010). Splicing of the *Xbp1* transcript is also required for adipogenesis (Sha et al., 2009).

### TMAO Reduces ER Stress and Lipid Accumulation in Adipocytes

To confirm that TMAO reduces ER stress and lipid metabolism, we tested these effects with 3T3-L1 adipocyte cell-based assays. We first assessed *Xbp1* splicing, because splicing of this central ER stress regulator is induced by ER stress and was validated as an ER stress marker recapitulating all other events in the IRE1a-XBP1 pathway (van Schadewijk et al., 2012). We confirmed that 0.1 mM TMAO inhibits tunicamycin-stimulated *Xbp1* splicing as efficiently as 0.1 mM 4-phenylbutyrate (PBA) (Figure 4C), an ER stress inhibitor also known to inhibit lipogenesis (Basseri et al., 2009). TMAO inhibits adipogenesis, as shown by decreased lipid accumulation (Figure 4D), which concurs with the negative association between TMAO excretion and the ratio of both EPD weight and EPD weight to body weight observed 20 days post-HFD (r = −0.302 and −0.291, respectively). These results show that TMAO, a known chemical chaperone (Ozcan et al., 2006), alleviates ER stress at 0.1 mM, as suggested by gene expression results, and impairs adipogenesis. Given that TMAO was also shown to reduce ER stress in β cells (Akerfeldt et al., 2008), we then tested the role of TMAO on glucose tolerance and insulin secretion in vivo in mice and in vitro in isolated islets.

### TMAO Improves Glucose Homeostasis and Insulin Secretion In Vivo

**Figure 5:**
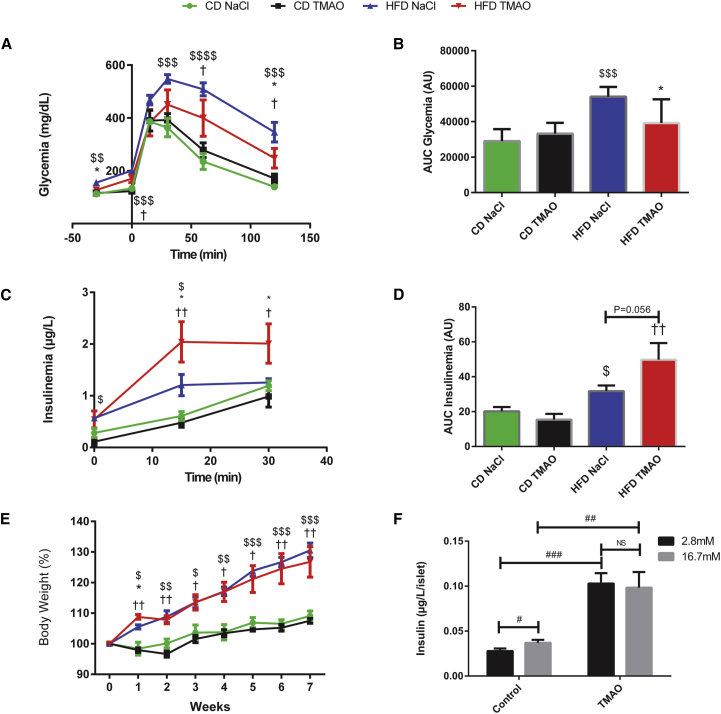
TMAO Partially Improves Glucose Tolerance on HFD through Increased Insulin Secretion (A) Plasma glucose profile during IP-GTT. (B) Cumulative glycemia (AUC). (C) Plasma insulin profile during IP-GTT. (D) Cumulative insulinemia (AUC). (E) Weekly BW monitoring. (F) Primary pancreatic islet insulin secretion. Glycemia and insulinemia were determined following an injection of glucose (2 g/kg BW) in control or HFD-fed mice treated by TMAO or NaCl for 5 weeks (n = 5 per group). BW was monitored throughout the duration of the experiment. The effect of TMAO on insulin production was tested in pancreatic islets cells (n = 20 mice, 1,705 islets in total) in response to 2.8 or 16.7 mM glucose. $p < 0.05 for control diet (CD) NaCl versus HFD NaCl, ^∗^p < 0.05 for HFD NaCl versus HFD TMAO, †p < 0.05 for HFD TMAO versus CD NaCl. #p < 0.05, ##p < 0.01, ###p < 0.001. Data are presented as means ± SEM.

To assess potential therapeutic effects of TMAO in vivo, we carried out glucose tolerance and insulin secretion tests in CHD- and HFD-fed mice treated by chronic subcutaneous infusion of this compound (Figure 5). HFD feeding resulted in significant elevation of fasting glycemia and insulinemia, glucose intolerance, enhanced insulin secretion induced by glucose, and increased BW after 7 weeks when compared to CHD-fed mice. TMAO infusion had no effect on glucose homeostasis, insulin secretion, or BW in CHD-fed mice (Figure 5E). In contrast, glucose tolerance (Figures 5A and 5B) was partially restored by chronic 6-week TMAO treatment in HFD-fed mice, as indicated by significant reduction of glycemia during the IP-GTT (Figure 5A) and cumulative glycemia during the test (Figure 5B) in TMAO-treated mice when compared to saline-treated controls. Improved glucose tolerance in TMAO-treated, HFD-fed mice was associated with further significant enhancement of insulin secretion response to glucose by TMAO in these mice (Figures 5C and 5D). We noted a tendency of TMAO to reduce body weight in mice fed HFD during the final 3 weeks of TMAO administration (Figure 5E), but differences were not statistically significant and the TMAO treatment could not be prolonged beyond 6 weeks for technical reasons. We then confirmed that TMAO directly increases insulin secretion in isolated islets (Figure 5F), which is consistent with its role as a chemical chaperone reducing ER stress in β cells (Akerfeldt et al., 2008). The partial IGT normalization induced by subcutaneous TMAO treatment in HFD-fed mice only suggests that TMAO’s beneficial role in the regulation of glucose homeostasis and insulin secretion is dependent of an interaction with diet.

## Discussion

Our results shed new light on metabolic roles of the gut microbiome in shaping host phenotypic heterogeneity and pre-disposition to disease susceptibility. Pre-intervention urinary metabotypes predict post-interventional disease outcomes and quantitative heterogeneity for a large number of traits (metabolic, hormonal, organ weights, and behavior) in isogenic mice. Microbial metabolites and their detoxification products belong to this prodromal signature, including microbial TMA and its product TMAO, which alleviates ER stress and reduces lipogenesis in cell-based assays, as well as improving glucose homeostasis by stimulating insulin production by pancreatic islets in vivo and in vitro.

### Microbiome-Driven Phenotypic Heterogeneity in Isogenic Mouse Populations

Variability of disease phenotypes in response to HFD in isogenic C57BL/6J mouse populations was proposed by Burcelin et al. (2002) as an alternative to diet-based comparisons; removing unwanted dietary confounders allowed the study of the role of microbial variations within this population (Serino et al., 2012). In our study, although the extreme groups were defined only on cumulative glycemia and BW, we observed a co-segregation of multiple traits, such as insulin secretion, blood lipids, organ weights, and behavior.

### Pre-dietary Intervention Metabolic Profiles Predict Disease Outcomes

By combining the isogenic mouse population model with a pharmaco-metabonomic approach, phenotypic heterogeneity in absence of genetic variation can be directly predicted by specific microbial-host co-metabolites, which could not be inferred through 16 s rRNA phylogenetic analysis (Serino et al., 2012). We show that pre-intervention urinary metabotypes predict post-intervention disease outcome and behavior patterns, thus showing that the pharmaco-metabonomics concept also applies to nutritional interventions and the prediction of disease risks or outcomes. The complexity of the metabolic patterns identified in our study suggests that each trait has a specific metabolic signature that is only similar to the signatures of other co-associated traits, which are best exemplified by BW, BMI, BW gain, and EPD and RFP weights or glycemic and insulinemic traits.

### Microbial-Host Co-metabolites Are Prodromal Markers of Phenotypic Heterogeneity

Several endogenous and microbial metabolites were identified in the predictive signatures for diabetes and obesity outcomes. In particular, TMAO, PAG, and hippurate are three microbial-mammalian co-metabolites obtained by phase 1 and phase 2 reactions in liver from their gut microbial substrates (TMA, phenylacetate, and benzoate, respectively). We observed that variations in baseline (i.e.. before HFD induction) excretion of TMAO and hippurate are strongly predictive of obesity risk. We previously showed that hippurate is negatively associated with BMI in humans (Elliott et al., 2015) and that benzoate variation was associated with a UGT2b polymorphism in the Goto-Kakizaki rat model of type 2 diabetes (Dumas et al., 2007). Post-intervention TMAO excretion was anti-correlated with obesity traits and was associated with a reduction of the expression of key enzymes involved in energy metabolism, lipid biosynthesis, and insulin signaling in adipose tissue. These observations support reports on the impact of the gut microbiome on brain development and anxiety behavior (Diaz Heijtz et al., 2011), while antibiotic therapy (Bercik et al., 2011) or *Lactobacillus* spp. supplementation (Bravo et al., 2011) affects behavior. In addition, *Bacteroides fragilis* affects the gut barrier in autism spectrum disorder mice, with circulating levels of microbial metabolites such as 4-ethylphenylsulfate eventually affecting their behavior (Hsiao et al., 2013).

### TMAO and Cardiometabolism

Methylamines represent the main microbial-mammalian co-metabolic pathway associated with HFD in our mouse model. TMAO results from a phase 1 *N*-oxidation of gut microbial TMA (al-Waiz et al., 1992) catabolized by FMO3 in humans (Dolphin et al., 1997). TMA is synthesized by gut microbial degradation of nitrogen-rich nutrients such as choline, phosphatidylcholine, and l-carnitine in decreasing order (al-Waiz et al., 1992, Russell et al., 2013). FMO3 is a target gene of the bile acid receptor FXR (Bennett et al., 2013) and was shown to play a central role in the regulation of cholesterol balance and glucose homeostasis (Warrier et al., 2015). This led to the establishment of a preliminary disease mechanism model in which TMAO and its dietary pre-cursors such as phosphatidylcholine and l-carnitine, found in red meat, could explain the increased cardiovascular disease (CVD) risk associated with red meat consumption (Koeth et al., 2013). While the association between TMAO and atherosclerosis initially reported by Tang et al. (2013) is now accepted (Bennett et al., 2013, Koeth et al., 2013, Koeth et al., 2014, Ussher et al., 2013, Wang et al., 2011b), the roles played by TMAO in glucose homeostasis are less clear (Bai et al., 1998, Gao et al., 2014, Lever et al., 2014, McEntyre et al., 2015).

### TMAO Alleviates ER Stress

Our results suggest that TMAO exposure reduces ER stress, resulting from the accumulation of misfolded proteins in the ER (Ozcan et al., 2006). This gene expression signature is particularly relevant because TMAO is an osmolyte acting as a chemical chaperone and stabilizing a three-dimensional protein structure, a role that was initially discovered in saltwater fish (Yancey et al., 1982). This general protein stabilization mechanism (Ma et al., 2014) is thought to reduce ER stress, which is involved in inflammation and insulin resistance (Ozcan et al., 2006), and could explain our observations for the role of TMAO on ER stress and *Xbp1* splicing in adipocytes. Similarly, TMAO corrects ER stress and *Xbp1* splicing induced by cytokines and palmitate in β cells (Akerfeldt et al., 2008).

### TMAO Infusion Improves Glucose Homeostasis, but Not Obesity, in Mice

Several conflicting studies exist about TMAO’s role on glucose homeostasis. The existing literature suggests that both dietary TMAO and FMO3 overexpression exacerbate IGT, whereas FMO3 is downregulated by insulin (Gao et al., 2014, Miao et al., 2015). To bypass indirect FMO3-specific effects (Bennett et al., 2013, Miao et al., 2015) triggered by potential microbial retroversion (Al-Waiz et al., 1987), we performed subcutaneous TMAO administration. The lack of effect of TMAO treatment on BW and BMI in HFD-fed mice supports the view that despite *Xbp1*’s central role in ER stress, adipocyte-specific *Xbp1* deletion does not affect obesity (Gregor et al., 2013). However, the marked improvement of HFD-induced IGT mediated by increased insulinemia, which we confirmed in vitro by treating isolated pancreatic islets with TMAO, is consistent with improved ER stress. Dietary TMAO was reported to exacerbate HFD-induced IGT after 3 weeks of feeding in male mice (Gao et al., 2014), whereas a previous report showed that subcutaneous or intraperitoneal TMAO injections lower glycemia (Bai et al., 1998). The beneficial effects of our 6-week subcutaneous TMAO infusion resulting in a partial correction of IGT through increased insulin secretion support the latter report. Our results are also consistent with the recent association of methylamines in general (and TMAO in particular) with the beneficial effects of *Akkermansia muciniphila* treatment in HFD-fed mice (Plovier et al., 2017). Analysis of long-term TMAO effects on β cell function and replication of the experiment in mice treated with oral administration of TMAO should improve our understanding of the impact of TMAO on glucose homeostasis and insulin secretion.

In conclusion, through extensive phenotyping, metabolomic, and transcriptomic studies, we show that microbial metabolites are prodromal markers and drivers of diet-induced phenotypic heterogeneity in isogenic mouse populations. We highlight a novel beneficial role for TMAO in glucose homeostasis and insulin secretion. Our work supports the emerging view that the gut microbiome can pre-dispose host health, opening perspectives in terms of predicting and monitoring functional effectiveness of dietary and microbiome interventions in stratified medicine.

## Experimental Procedures

### Animals

All experiments were approved by the ethical committees of the University of Oxford and University Pierre & Marie Curie. Male C57BL/6J mice were bred in the laboratory. All mice were kept under standard maintenance conditions on 12 hr light/dark cycle.

### Heterogeneous Mouse Populations

Mice were weaned at 21 days and caged in groups of ten throughout the whole experiment. Mice were fed a normal carbohydrate (CHD) diet containing 5% fat, 19% protein, and 3.5% fiber (w/w). At 5 weeks, a group of mice (n = 50–193) was transferred to a 40% w/w (65% kcal) (HFD) ad libitum, while a group of age-matched mice remained on CHD throughout the experiment as described previously (Dumas et al., 2006, Fearnside et al., 2008). Blood and urine samples were collected in both groups. Metabolic homeostasis was assessed by glucose tolerance tests after 3 weeks of treatment. Animals were then killed by CO_2_ asphyxiation. Tissues were collected, weighed, and snap-frozen in liquid nitrogen.

### TMAO Infusion

Six-week-old C57BL/6J mice were fed a standard CHD, and at 9 weeks, a group of ten mice was transferred to a HFD. At 10 weeks, osmotic minipumps were inserted subcutaneously in mice under ketamine-xylazine anesthesia to deliver NaCl or TMAO (2.78 mM in 0.9% NaCl) for 6 weeks as described previously (Cani et al., 2007).

### Insulin Secretion from Isolated Islets

Six-week-old C57BL/6J male mice (n = 20) were euthanized by cervical dislocation and pancreatic islets isolated by collagenase digestion. Groups of 5–6 islets per well (1,705 islets in total) were incubated in presence of TMAO in culture media. Islets were then incubated in 2.8 mM glucose to measure basal insulin production and subsequently in 16.7 mM glucose to measure glucose stimulated insulin secretion.

### ER Stress

ER stress was assessed in 3T3-L1 adipocytes after 7 days of differentiation, upon ER stress stimulation by 50 ng/mL tunicamycin, and rescued by 10 mM PBA or 10 mM TMAO. RNA was extracted from cells, and differential splicing of XBP1 mRNA was assessed by qPCR before reverse transcription using previously described primers for total XBP1 (XBP1t) and spliced XBP1 (XBP1s) (Wang et al., 2011a).

### Lipid Accumulation

Oil red O staining was performed in differentiated 3T3-L1 adipocytes after 9 days of differentiation. Oil red O was added for 10 min. The dye bound to lipids was resuspended using isopropanol, and optical density at 520 nm was read on a spectrophotometer. The oil red O quantification was then normalized to cell viability assessed by crystal violet staining for 30 min, resuspended in methanol, and read at 600 nm.

### Glucose Tolerance and Insulin Secretion Tests

Body weight (BW) was recorded and intraperitoneal glucose tolerance tests (IP-GTT, 2 g/kg BW) were performed in overnight-fasted mice as previously described (Fearnside et al., 2008).

### Behavioral Tests

EPM and OF were used to assess rodent exploration, activity, and anxiety as previously described (Solberg et al., 2006, Valdar et al., 2006). Animals were all naively tested at 8 weeks of age.

### Transcriptomics

Experiments were performed according to Affymetrix protocols as previously described (Toye et al., 2007). Microarray data were analyzed using R and the Bioconductor packages affy (Gautier et al., 2004), LIMMA (linear models for microarray data) (Smyth, 2005), and BiNGO (Maere et al., 2005).

### Metabolic Phenotyping

Urine samples were profiled using a ^1^H-NMR spectrometer operating at a 600.22 MHz ^1^H frequency, and spectra were imported into MATLAB (R2012b, MathWorks) as described previously (Dumas et al., 2006). The dataset was then further aligned using recursive segment-wise peak alignment (RSPA) (Veselkov et al., 2009), and peak calling was performed using statistical recoupling of variables (SRVs) (Blaise et al., 2009). Variance-stabilizing logarithmic transform of the SRV clusters (Veselkov et al., 2011) and probabilistic quotient normalization (Dieterle et al., 2006) were used before multivariate analyses. Predictive models were built using O-PLS-DA with 7-fold cross-validation. Models were validated by permutations testing of the Q^2^_Yhat_ goodness-of-prediction statistics parameter with 10,000 random iterations and calculation of an empirical p value (Blaise et al., 2007).

Experiments are Minimum Information About a Microarray Experiment (MIAME) compliant. See Supplemental Experimental Procedures for more details.

## Author Contributions

Conceptualization, M.-E.D., J.K.N., and D.G.; Investigation, M.-E.D., A.R.R., J.C., S.C., C.H., C.D., R.H.B., T.A., J.F.F., C.B., and N.P.; Analysis, M.-E.D., A.R.R., L.H., Q.G., E.M.N., and C.L.B.; Supervision, M.-E.D., J.K.N., and D.G.; Writing – Original Draft, M.-E.D., A.R.R., and D.G.; Writing – Review and Editing, M.-E.D., J.K.N., and D.G.; Funding Acquisition, M.-E.D., J.S., J.K.N., and D.G.

## Accession Numbers

The accession number for the full protocols and data reported in this paper is ArrayExpress: E-MTAB-2569.

## Supplemental Information

Document S1. Supplemental Experimental Procedures and Figures S1–S4

Table S1. Metabolite Assignment by ^1^H-NMR Spectroscopy, Related to Figure 3

Table S2. Adipose Tissue Transcripts Significantly Correlated with TMAO, Related to Figure 4

Table S3. Gene Ontology Analysis of TMAO-Correlated Genes, Related to Figure 4

Table S4. Distribution of TMAO-Correlated Genes in Relevant KEGG Pathways, Related to Figure 4

Document S2. Article plus Supplemental Information

## References

[bib1] Akerfeldt M.C., Howes J., Chan J.Y., Stevens V.A., Boubenna N., McGuire H.M., King C., Biden T.J., Laybutt D.R. (2008). Cytokine-induced beta-cell death is independent of endoplasmic reticulum stress signaling. Diabetes.

[bib2] Al-Waiz M., Ayesh R., Mitchell S.C., Idle J.R., Smith R.L. (1987). Disclosure of the metabolic retroversion of trimethylamine *N*-oxide in humans: a pharmacogenetic approach. Clin. Pharmacol. Ther..

[bib3] al-Waiz M., Mikov M., Mitchell S.C., Smith R.L. (1992). The exogenous origin of trimethylamine in the mouse. Metabolism.

[bib4] Bai C., Biwersi J., Verkman A.S.A., Matthay M.A.M. (1998). A mouse model to test the in vivo efficacy of chemical chaperones. J. Pharmacol. Toxicol. Methods.

[bib5] Basseri S., Lhoták S., Sharma A.M., Austin R.C. (2009). The chemical chaperone 4-phenylbutyrate inhibits adipogenesis by modulating the unfolded protein response. J. Lipid Res..

[bib6] Bennett B.J., de Aguiar Vallim T.Q., Wang Z., Shih D.M., Meng Y., Gregory J., Allayee H., Lee R., Graham M., Crooke R. (2013). Trimethylamine-*N*-oxide, a metabolite associated with atherosclerosis, exhibits complex genetic and dietary regulation. Cell Metab..

[bib7] Bercik P., Denou E., Collins J., Jackson W., Lu J., Jury J., Deng Y., Blennerhassett P., Macri J., McCoy K.D. (2011). ). The intestinal microbiota affect central levels of brain-derived neurotropic factor and behavior in mice. Gastroenterology.

[bib8] Blaise B.J., Giacomotto J., Elena B., Dumas M.-E., Toulhoat P., Ségalat L., Emsley L. (2007). Metabotyping of *Caenorhabditis elegans* reveals latent phenotypes. Proc. Natl. Acad. Sci. USA.

[bib9] Blaise B.J., Shintu L., Elena B., Emsley L., Dumas M.-E., Toulhoat P. (2009). Statistical recoupling prior to significance testing in nuclear magnetic resonance based metabonomics. Anal. Chem..

[bib10] Bravo J.A., Forsythe P., Chew M.V., Escaravage E., Savignac H.M., Dinan T.G., Bienenstock J., Cryan J.F. (2011). Ingestion of *Lactobacillus* strain regulates emotional behavior and central GABA receptor expression in a mouse via the vagus nerve. Proc. Natl. Acad. Sci. USA.

[bib11] Burcelin R., Crivelli V., Dacosta A., Roy-Tirelli A., Thorens B. (2002). Heterogeneous metabolic adaptation of C57BL/6J mice to high-fat diet. Am. J. Physiol. Endocrinol. Metab..

[bib12] Cani P.D., Amar J., Iglesias M.A., Poggi M., Knauf C., Bastelica D., Neyrinck A.M., Fava F., Tuohy K.M., Chabo C. (2007). Metabolic endotoxemia initiates obesity and insulin resistance. Diabetes.

[bib13] Clayton T.A., Lindon J.C., Cloarec O., Antti H., Charuel C., Hanton G., Provost J.-P., Le Net J.-L., Baker D., Walley R.J. (2006). Pharmaco-metabonomic phenotyping and personalized drug treatment. Nature.

[bib14] Cotillard A., Kennedy S.P., Kong L.C., Prifti E., Pons N., Le Chatelier E., Almeida M., Quinquis B., Levenez F., Galleron N., ANR MicroObes consortium (2013). Dietary intervention impact on gut microbial gene richness. Nature.

[bib15] Craciun S., Balskus E.P. (2012). Microbial conversion of choline to trimethylamine requires a glycyl radical enzyme. Proc. Natl. Acad. Sci. USA.

[bib16] Dao M.C., Everard A., Aron-Wisnewsky J., Sokolovska N., Prifti E., Verger E.O., Kayser B.D., Levenez F., Chilloux J., Hoyles L., MICRO-Obes Consortium (2016). *Akkermansia muciniphila* and improved metabolic health during a dietary intervention in obesity: relationship with gut microbiome richness and ecology. Gut.

[bib17] David L.A., Maurice C.F., Carmody R.N., Gootenberg D.B., Button J.E., Wolfe B.E., Ling A.V., Devlin A.S., Varma Y., Fischbach M.A. (2014). Diet rapidly and reproducibly alters the human gut microbiome. Nature.

[bib18] Diaz Heijtz R., Wang S., Anuar F., Qian Y., Björkholm B., Samuelsson A., Hibberd M.L., Forssberg H., Pettersson S. (2011). Normal gut microbiota modulates brain development and behavior. Proc. Natl. Acad. Sci. USA.

[bib19] Dieterle F., Ross A., Schlotterbeck G., Senn H. (2006). Probabilistic quotient normalization as robust method to account for dilution of complex biological mixtures. Application in ^1^H NMR metabonomics. Anal. Chem..

[bib20] Dolphin C.T., Janmohamed A., Smith R.L., Shephard E.A., Phillips I.R. (1997). Missense mutation in flavin-containing mono-oxygenase 3 gene, FMO3, underlies fish-odour syndrome. Nat. Genet..

[bib21] Dumas M.-E. (2011). The microbial-mammalian metabolic axis: beyond simple metabolism. Cell Metab..

[bib22] Dumas M.-E., Barton R.H., Toye A., Cloarec O., Blancher C., Rothwell A., Fearnside J., Tatoud R., Blanc V., Lindon J.C. (2006). Metabolic profiling reveals a contribution of gut microbiota to fatty liver phenotype in insulin-resistant mice. Proc. Natl. Acad. Sci. USA.

[bib23] Dumas M.-E., Wilder S.P., Bihoreau M.-T., Barton R.H., Fearnside J.F., Argoud K., D’Amato L., Wallis R.H., Blancher C., Keun H.C. (2007). Direct quantitative trait locus mapping of mammalian metabolic phenotypes in diabetic and normoglycemic rat models. Nat. Genet..

[bib24] Dumas M.-E., Kinross J., Nicholson J.K. (2014). Metabolic phenotyping and systems biology approaches to understanding metabolic syndrome and fatty liver disease. Gastroenterology.

[bib25] Elliott P., Posma J.M., Chan Q., Garcia-Perez I., Wijeyesekera A., Bictash M., Ebbels T.M., Ueshima H., Zhao L., van Horn L. (2015). Urinary metabolic signatures of human adiposity. Sci. Transl. Med..

[bib26] Fearnside J.F., Dumas M.-E., Rothwell A.R., Wilder S.P., Cloarec O., Toye A., Blancher C., Holmes E., Tatoud R., Barton R.H. (2008). Phylometabonomic patterns of adaptation to high fat diet feeding in inbred mice. PLoS ONE.

[bib27] Gao X., Liu X., Xu J., Xue C., Xue Y., Wang Y. (2014). Dietary trimethylamine *N*-oxide exacerbates impaired glucose tolerance in mice fed a high fat diet. J. Biosci. Bioeng..

[bib28] Gautier L., Cope L., Bolstad B.M., Irizarry R.A. (2004). affy—analysis of Affymetrix GeneChip data at the probe level. Bioinformatics.

[bib29] Gavaghan C.L., Holmes E., Lenz E., Wilson I.D., Nicholson J.K. (2000). An NMR-based metabonomic approach to investigate the biochemical consequences of genetic strain differences: application to the C57BL10J and Alpk:ApfCD mouse. FEBS Lett..

[bib30] Gregor M.F., Misch E.S., Yang L., Hummasti S., Inouye K.E., Lee A.-H., Bierie B., Hotamisligil G.S. (2013). The role of adipocyte XBP1 in metabolic regulation during lactation. Cell Rep..

[bib31] Hotamisligil G.S. (2010). Endoplasmic reticulum stress and the inflammatory basis of metabolic disease. Cell.

[bib32] Hsiao E.Y., McBride S.W., Hsien S., Sharon G., Hyde E.R., McCue T., Codelli J.A., Chow J., Reisman S.E., Petrosino J.F. (2013). Microbiota modulate behavioral and physiological abnormalities associated with neurodevelopmental disorders. Cell.

[bib33] Karlsson F.H., Tremaroli V., Nookaew I., Bergström G., Behre C.J., Fagerberg B., Nielsen J., Bäckhed F. (2013). Gut metagenome in European women with normal, impaired and diabetic glucose control. Nature.

[bib34] Koeth R.A., Wang Z., Levison B.S., Buffa J.A., Org E., Sheehy B.T., Britt E.B., Fu X., Wu Y., Li L. (2013). Intestinal microbiota metabolism of L-carnitine, a nutrient in red meat, promotes atherosclerosis. Nat. Med..

[bib35] Koeth R.A., Levison B.S., Culley M.K., Buffa J.A., Wang Z., Gregory J.C., Org E., Wu Y., Li L., Smith J.D. (2014). γ-Butyrobetaine is a proatherogenic intermediate in gut microbial metabolism of L-carnitine to TMAO. Cell Metab..

[bib36] Le Chatelier E., Nielsen T., Qin J., Prifti E., Hildebrand F., Falony G., Almeida M., Arumugam M., Batto J.-M., Kennedy S., MetaHIT consortium (2013). Richness of human gut microbiome correlates with metabolic markers. Nature.

[bib37] Lehner B. (2013). Genotype to phenotype: lessons from model organisms for human genetics. Nat. Rev. Genet..

[bib38] Lever M., George P.M., Slow S., Bellamy D., Young J.M., Ho M., McEntyre C.J., Elmslie J.L., Atkinson W., Molyneux S.L. (2014). Betaine and trimethylamine-*N*-oxide as predictors of cardiovascular outcomes show different patterns in diabetes mellitus: an observational study. PLoS ONE.

[bib39] Li J., Jia H., Cai X., Zhong H., Feng Q., Sunagawa S., Arumugam M., Kultima J.R., Prifti E., Nielsen T., MetaHIT Consortium (2014). An integrated catalog of reference genes in the human gut microbiome. Nat. Biotechnol..

[bib40] Ma J., Pazos I.M., Gai F. (2014). Microscopic insights into the protein-stabilizing effect of trimethylamine *N*-oxide (TMAO). Proc. Natl. Acad. Sci. USA.

[bib41] Maere S., Heymans K., Kuiper M. (2005). BiNGO: a Cytoscape plugin to assess overrepresentation of gene ontology categories in biological networks. Bioinformatics.

[bib42] McEntyre C.J., Lever M., Chambers S.T., George P.M., Slow S., Elmslie J.L., Florkowski C.M., Lunt H., Krebs J.D. (2015). Variation of betaine, *N*,*N*-dimethylglycine, choline, glycerophosphorylcholine, taurine and trimethylamine-*N*-oxide in the plasma and urine of overweight people with type 2 diabetes over a two-year period. Ann. Clin. Biochem..

[bib43] Miao J., Ling A.V., Manthena P.V., Gearing M.E., Graham M.J., Crooke R.M., Croce K.J., Esquejo R.M., Clish C.B., Vicent D., Biddinger S.B., Morbid Obesity Study Group (2015). Flavin-containing monooxygenase 3 as a potential player in diabetes-associated atherosclerosis. Nat. Commun..

[bib44] Muegge B.D., Kuczynski J., Knights D., Clemente J.C., González A., Fontana L., Henrissat B., Knight R., Gordon J.I. (2011). Diet drives convergence in gut microbiome functions across mammalian phylogeny and within humans. Science.

[bib45] Nicholson J.K., Holmes E., Kinross J., Burcelin R., Gibson G., Jia W., Pettersson S. (2012). Host-gut microbiota metabolic interactions. Science.

[bib46] Ozcan U., Yilmaz E., Ozcan L., Furuhashi M., Vaillancourt E., Smith R.O., Görgün C.Z., Hotamisligil G.S. (2006). Chemical chaperones reduce ER stress and restore glucose homeostasis in a mouse model of type 2 diabetes. Science.

[bib47] Plovier H., Everard A., Druart C., Depommier C., Van Hul M., Geurts L., Chilloux J., Ottman N., Duparc T., Lichtenstein L. (2017). A purified membrane protein from *Akkermansia muciniphila* or the pasteurized bacterium improves metabolism in obese and diabetic mice. Nat. Med..

[bib48] Qin J., Li Y., Cai Z., Li S., Zhu J., Zhang F., Liang S., Zhang W., Guan Y., Shen D. (2012). A metagenome-wide association study of gut microbiota in type 2 diabetes. Nature.

[bib49] Ridaura V.K., Faith J.J., Rey F.E., Cheng J., Duncan A.E., Kau A.L., Griffin N.W., Lombard V., Henrissat B., Bain J.R. (2013). Gut microbiota from twins discordant for obesity modulate metabolism in mice. Science.

[bib50] Russell W.R., Hoyles L., Flint H.J., Dumas M.-E. (2013). Colonic bacterial metabolites and human health. Curr. Opin. Microbiol..

[bib51] Serino M., Luche E., Gres S., Baylac A., Bergé M., Cenac C., Waget A., Klopp P., Iacovoni J., Klopp C. (2012). Metabolic adaptation to a high-fat diet is associated with a change in the gut microbiota. Gut.

[bib52] Sha H., He Y., Chen H., Wang C., Zenno A., Shi H., Yang X., Zhang X., Qi L. (2009). The IRE1alpha-XBP1 pathway of the unfolded protein response is required for adipogenesis. Cell Metab..

[bib53] Shoaie S., Ghaffari P., Kovatcheva-Datchary P., Mardinoglu A., Sen P., Pujos-Guillot E., de Wouters T., Juste C., Rizkalla S., Chilloux J., MICRO-Obes Consortium (2015). Quantifying diet-induced metabolic changes of the human gut microbiome. Cell Metab..

[bib54] Smith M.I., Yatsunenko T., Manary M.J., Trehan I., Mkakosya R., Cheng J., Kau A.L., Rich S.S., Concannon P., Mychaleckyj J.C. (2013). Gut microbiomes of Malawian twin pairs discordant for kwashiorkor. Science.

[bib55] Smyth G.K., Gentleman R., Carey V.J., Huber W., Irizarry R.A., Dudoit S. (2005). limma: linear models for microarray data. Bioinformatics and Computational Biology Solutions Using R and Bioconductor.

[bib56] Solberg L.C., Valdar W., Gauguier D., Nunez G., Taylor A., Burnett S., Arboledas-Hita C., Hernandez-Pliego P., Davidson S., Burns P. (2006). A protocol for high-throughput phenotyping, suitable for quantitative trait analysis in mice. Mamm. Genome.

[bib57] Tang W.H.W., Wang Z., Levison B.S., Koeth R.A., Britt E.B., Fu X., Wu Y., Hazen S.L. (2013). Intestinal microbial metabolism of phosphatidylcholine and cardiovascular risk. N. Engl. J. Med..

[bib58] Toye A.A., Dumas M.E., Blancher C., Rothwell A.R., Fearnside J.F., Wilder S.P., Bihoreau M.T., Cloarec O., Azzouzi I., Young S. (2007). Subtle metabolic and liver gene transcriptional changes underlie diet-induced fatty liver susceptibility in insulin-resistant mice. Diabetologia.

[bib59] Turnbaugh P.J., Ley R.E., Mahowald M.A., Magrini V., Mardis E.R., Gordon J.I. (2006). An obesity-associated gut microbiome with increased capacity for energy harvest. Nature.

[bib60] Ussher J.R., Lopaschuk G.D., Arduini A. (2013). Gut microbiota metabolism of L-carnitine and cardiovascular risk. Atherosclerosis.

[bib61] Valdar W., Solberg L.C., Gauguier D., Cookson W.O., Rawlins J.N.P., Mott R., Flint J. (2006). Genetic and environmental effects on complex traits in mice. Genetics.

[bib62] van Schadewijk A., van’t Wout E.F.A., Stolk J., Hiemstra P.S. (2012). A quantitative method for detection of spliced X-box binding protein-1 (XBP1) mRNA as a measure of endoplasmic reticulum (ER) stress. Cell Stress Chaperones.

[bib63] Venkatesh M., Mukherjee S., Wang H., Li H., Sun K., Benechet A.P., Qiu Z., Maher L., Redinbo M.R., Phillips R.S. (2014). Symbiotic bacterial metabolites regulate gastrointestinal barrier function via the xenobiotic sensor PXR and Toll-like receptor 4. Immunity.

[bib64] Veselkov K.A., Lindon J.C., Ebbels T.M.D., Crockford D., Volynkin V.V., Holmes E., Davies D.B., Nicholson J.K. (2009). Recursive segment-wise peak alignment of biological (1)h NMR spectra for improved metabolic biomarker recovery. Anal. Chem..

[bib65] Veselkov K.A., Vingara L.K., Masson P., Robinette S.L., Want E., Li J.V., Barton R.H., Boursier-Neyret C., Walther B., Ebbels T.M. (2011). Optimized preprocessing of ultra-performance liquid chromatography/mass spectrometry urinary metabolic profiles for improved information recovery. Anal. Chem..

[bib66] Wang F.-M., Chen Y.-J., Ouyang H.-J. (2011). Regulation of unfolded protein response modulator XBP1s by acetylation and deacetylation. Biochem. J..

[bib67] Wang Z., Klipfell E., Bennett B.J., Koeth R., Levison B.S., Dugar B., Feldstein A.E., Britt E.B., Fu X., Chung Y.-M. (2011). Gut flora metabolism of phosphatidylcholine promotes cardiovascular disease. Nature.

[bib68] Warrier M., Shih D.M., Burrows A.C., Ferguson D., Gromovsky A.D., Brown A.L., Marshall S., McDaniel A., Schugar R.C., Wang Z. (2015). The generating enzyme flavin monooxygenase 3 is a central regulator of cholesterol balance. Cell Rep..

[bib69] Yancey P.H., Clark M.E., Hand S.C., Bowlus R.D., Somero G.N. (1982). Living with water stress: evolution of osmolyte systems. Science.

[bib70] Yoshimoto S., Loo T.M., Atarashi K., Kanda H., Sato S., Oyadomari S., Iwakura Y., Oshima K., Morita H., Hattori M. (2013). Obesity-induced gut microbial metabolite promotes liver cancer through senescence secretome. Nature.

