# Supplementary material for: Microbial-Host Co-metabolites Are Prodromal Markers Predicting Phenotypic Heterogeneity in Behavior, Obesity, and Impaired Glucose Tolerance
Source: Cell Rep. 2017 Jul 5;20(1):136–48. doi: 10.1016/j.celrep.2017.06.039 (PMC5507771; doi:10.1016/j.celrep.2017.06.039)
Supplement: Document S1. Supplemental Experimental Procedures and Figures S1–S4 [file mmc1.pdf]

Cell Reports, Volume *20*

Supplemental Information

Microbial-Host Co-metabolites Are Prodromal Markers Predicting Phenotypic Heterogeneity in Behavior, Obesity, and Impaired Glucose Tolerance

Marc-Emmanuel Dumas, Alice R. Rothwell, Lesley Hoyles, Thomas Aranias, Julien Chilloux, Sophie Calderari, Elisa M. Noll, Noémie Péan, Claire L. Boulangé, Christine Blancher, Richard H. Barton, Quan Gu, Jane F. Fearnside, Chloé Deshayes, Christophe Hue, James Scott, Jeremy K. Nicholson, and Dominique Gauguier

SUPPLEMENTAL EXPERIMENTAL PROCEDURES

Animals. Male C57BL/6J mice were bred in the laboratory. All mice were kept under standard maintenance conditions on 12 h light/dark cycle.

Heterogeneous mouse populations. Mice were weaned at 21 days and caged in groups of 10 throughout the entire experiments. They were fed a normal carbohydrate (CHD) diet containing 5 % fat, 19 % protein and 3.5 % fibre (S&K Universal Ltd, Hull, UK). At five weeks, a group of 50 mice was transferred to a 40 %w/w (65%Kcal) high fat diet (HFD) containing 32 % pig fat, 8 % casein fat, 19 % protein, 21 % glucose (Special Diet Services, Amersham, UK) *ad libitum*. A group of 50 age-matched mice remained on CHD throughout the experiment. All experiments were carried out under licences granted by the UK Home Office and approved by the ethical committee of the University of Oxford.

TMAO infusion. Six-week old C57BL/6J mice purchased from a commercial supplier (Charles River, L'Arbresle, France) were maintained in specific pathogen-free (SPF) conditions. Mice were fed a standard chow diet and at nine weeks, a group of 10 mice was transferred to a HFD (respectively D12450Ki and D12492i, Research diets, New Brunswick, NJ), as below.

General Composition	HFD (40%w/w, 65%Kcal)	CHD (5% w/w
Crude oil	38.96	4.73
Crude protein	20.28	18.68
Crude fiber	3.5	3.48
Ash	2.88	5.38
NFE	23.86	59.73

At ten weeks, mice were anaesthetised by injection of ketamine-xylazine (Centravet, Plancoet, France) for subcutaneous implantation of osmotic minipumps (Alzet® 2006, Charles River Lab France, L'Arbresle, France) to deliver either NaCl or TMAO for a period to 6 weeks, as described previously (Cani et al., 2007). Minipumps were originally primed according to the manufacturer's instructions and filled with solutions of either 0.9% NaCl or 2.78 mM TMAO in 0.9% NaCl. Fed blood glucose and BW were measured every week. After five weeks of NaCl or TMAO treatment, an intra-peritoneal glucose tolerance and insulin secretion test (2g glucose/kg BW) was performed in conscious mice. Blood glucose was determined with an Accu-Chek® Performa (Roche Diagnostics, Meylan, France). Paralleled blood samples were taken to determine plasma concentration of insulin using an ELISA kit (Mercodia, Uppsala, Sweden).

Insulin secretion from isolated pancreatic islets. Six-week old C57BL/6J male mice (n=20) purchased from a commercial supplier (Charles River, L'Arbresle, France) were euthanized by cervical dislocation and pancreatic islets isolated by collagenase digestion (Boerhinger Mannheim, Mannheim, Germany). Groups of 5-6 islets (1705 islets in total) per well were incubated in presence of TMAO in a culture medium consisting of RPMI 1640, 11

mM glucose, 100 units/mL penicillin, 100 µg/mL streptomycin, 40 µg/mL gentamycin and 10% FCS. Islets were then incubated in 2.8 mM glucose to measure basal insulin production and subsequently in 16.7 mM glucose to measure glucose stimulated insulin secretion. A total of 6 independent experiments were carried out.

Glucose tolerance and insulin secretion tests. Body weight (BW) was recorded and intraperitoneal glucose tolerance tests (IP-GTT, 2 g/kg BW) were performed in overnight-fasted mice as previously described (Fearnside et al., 2008). Blood samples were collected from the tip of the tail vein before the injection and 15, 30 and 75 min afterward. Blood glucose concentration was determined immediately. Cumulative glycemia (CumG) was calculated as the increment of plasma glucose values during the test. Plasma samples were separated by centrifugation and stored at -80 °C until insulin assay. CumG and cumulative insulinemia (CumIRI) were calculated as the increment of the values of plasma glucose and insulin, respectively, during the test. Data from the IP-GTT was used to determine the homeostasis model assessment of insulin resistance (HOMA).

Biofluid collection and tissue sampling. Blood and urine samples were collected in mice of the CHD and HFD groups at 5 weeks of age (before the HFD induction for the HFD group) and at 2 and 5 months of age (before the IP-GTT). BW and body length (BL) were determined and body mass index (BMI) was calculated as the ratio BW/BL^2. Mice were housed individually in metabolic cages (Techniplast, Italy) for a 48-h period before the sampling. Animals were then killed by CO_2 asphyxiation. Brown adipose tissue and retroperitoneal and epididymal fat pads (RFP and EPD, respectively) were collected, weighed and snap-frozen in liquid nitrogen. Adiposity index (AI) was calculated as the ratio between RFP or EPD weight to BW (AI_RFP and AI_EPD, respectively).

Clinical biochemistry. Blood glucose levels were determined with a glucose meter (Accucheck, Roche Diagnostics). Plasma immunoreactive insulin (IRI) was determined with an ELISA kit (Mercodia, Uppsala, Sweden). Plasma concentrations of triglycerides and total, HDL and LDL cholesterol were determined using diagnostic enzymatic/colorimetric kits (ABX, Shefford, UK) on a Cobas Mira Plus automatic analyser (ABX, Shefford, UK).

Behavioural Tests. Elevated Plus Maze (EPM) and Open Field (OF) were used to assess rodent exploration, activity and anxiety as previously described (Solberg et al., 2006; Valdar et al., 2006). Animals were all naïvely tested at 8 weeks of age.

EPM. The EPM platform was 1 m above floor level, with a grey-coloured surface at least 1 m radius of free space around the maze, illuminated from an overhead source (1 m above the platform with an aluminium shade) at 100 Lux intensity. All tests were 5 min in duration. All mice were first placed in the centre, facing the same open arm. Activity was scored as the number of entries into either the closed or open arms as well as number of entries into the centre (total entries). Anxiety was measured as relative time spent in each of the compartments.

Open field (OF). OF tests were performed in a clear rectangular plastic four-walled container (54.5 cm x 32.5 cm) set in the middle of the floor in the same room used for the EPM tests. White grid lines were marked (producing squares 11 cm x 11 cm) on a black sheet of cardboard, which was laid under the box. Each trial lasted for 3 min. Activity was measured by the number of transitions into different squares. The number of rearings was also recorded to quantify activity. The grid divided the area into outer or inner squares. Mice were always placed in the same middle outer square on one of the ends of the rectangle, and time taken to enter the 'inner' arena was recorded as 'the latency to enter the centre'. Relative time spent in these inner and outer compartments was also calculated.

Cell-Based Assays.

Insulin secretion in isolated islets. Insulin secreted in cell medium was measured using an ELISA kit (Mercodia, Uppsala, Sweden).

Adipocyte differentiation. 3T3-L1 fibroblasts cells (ATCC, Molsheim, France) were routinely cultured in DMEM high glucose (Invitrogen, St Aubin, France) containing 10 % Calf Serum (Life Technology, St Aubin, France) at 37 °C, 5 % CO_2. To initiate adipocyte differentiation, 3T3-L1 fibroblasts were plated in P24 dishes (Becton Dickinson, Courtaboeuf, France) at a density of 10^4 cells/dish. At this density, cells reached confluence 5 days later and then the medium was replaced. After 48h (day 0), adipocyte differentiation was induced by changing the medium to DMEM high glucose containing 10% fetal bovine serum (PAA, Les Mureaux, France), 0.5 mM IBMX (Sigma Aldrich, St Quentin, France), 0.25 μM dexamethasone (Sigma Aldrich, St Quentin, France) and 0.4 μM insulin (Sigma Aldrich, St Quentin, France). After 72 h (day 3), the medium was changed to DMEM high glucose containing 10% FBS, and 0.4 μM insulin. After 48 h (day 5), the medium was changed as at D3. At D7, adipocytes were maintained in DMEM high glucose with 5 % FBS and refreshed every 2 days.

Metabolite supplementation. TMAO (Sigma Aldrich, St Quentin, France) was added either during the differentiation or at the end of adipocyte differentiation. Three concentrations of the metabolite (1 M, 10 mM and 0.1 mM, diluted in water) were tested in quadruplet. To test the impact of metabolite supplementation during adipocyte differentiation, metabolites were added at day 0, 3 and 5.

Endoplasmic Reticulum (ER) stress assay. ER stress was assessed in 3T3-L1 cells after 7 days of differentiation, upon ER stress stimulation by 50 ng/mL tunicamycin and rescued by 10 mM 4-phenylbutyrate or 10 mM TMAO (Sigma Aldrich, Gillingham, UK). RNA was extracted from cells using RNeasy Mini Kit (Qiagen, Manchester, UK). Reverse transcription was performed on 500ng RNA using SuperScript II Reverse Transcriptase (Invitrogen) following provided instructions. Differential splicing of XBP1 mRNA was quantified by quantitative PCR (qPCR) on a StepOnePlus system using SYBR Select Master Mix (Life Technologies, Paisley, UK) and previously described primers for total XBP1 (XBP1t) and spliced XBP1 (XBP1s) as follows (Wang et al., 2011): XBP1t fw: 5'-AAGAACACGCTTGGGAATGG-3', XBP1t rv: 5'-ACTCCCCTTGGCCTCCAC-3', XBP1s fw:

5'-GAGTCCGCAGCAGGTG-3' and XBP1s rv: 5'-GTGTCAGAGTCCATGGGA-3'. Results are presented as the ratio of XBP1s to XBP1t expression determined by the relative quantification method (change in cycle threshold).

Lipid uptake assay. Oil Red-O staining was performed at day 7. Oil Red-O stock solution was prepared by stirring 0.35 % Oil Red-O (Sigma Aldrich, St Quentin, France) in isopropanol overnight followed by filtration (0.2 µm). Oil Red O working solution was prepared by mixing stock solution with distilled water (6:4), followed by incubation for 10 min at room temperature. Cells were incubated 5 min with 500 µL 10 % formaldehyde (Sigma Aldrich, St Quentin, France) and fixed with 500 µL 10 % formaldehyde at least 1 h at room temperature. Subsequently, the cells were washed once with 60 % isopropanol and dried. Oil Red-O working solution (200 µL/dish P24) was added for 10 min. Dishes were then washed four times with distilled water before analysis. After microscopic photography by light transmission, the oil-red-O dye bound to lipids was suspended using isopropanol, and quantifications were obtained by reading the optical density at 500 nm on a spectrophotometer (Biotek).

Glucose uptake assay. Glucose uptake in differentiating adipocytes was carried out to test the impact of metabolite supplementation on adipocyte function. After 4h in DMEM high glucose without FBS (Life Technology, St Aubin, France), adipocytes were incubated for 20 min with insulin (100nM) and then 20 min with 0.5 microCi 2{^{3}H}DeoxyD-Glucose. Cells were then collected in NaOH 300 mM and the incorporated radioactivity was measured.

Gene transcription profiling. Total RNA form liver of six mice per group was extracted using Trizol reagent (Invitrogen Life Technologies, Paisley, UK) and cleaned with RNeasy columns (Qiagen Ltd., Crawley, UK). RNA concentrations and integrity were assessed using an Agilent 2100 Bioanalyser (Agilent Technologies, Waldbronn, Germany). RNA probes were hybridized to Affymetrix arrays U430 2.0 (Affymetrix UK ltd, High Wycombe, UK) containing 45,266 probesets, respectively, and allowing quantification of the abundance of transcripts corresponding to 20,827 independent gene and EST sequences. Experiments were performed according to Affymetrix protocols as previously described (Toye et al., 2007). Experiments are MIAME compliant and full protocols and data are publicly available (www.ebi.ac.uk/arrayexpress/) under the accession E-MTAB-2569.

^{1}H NMR Spectroscopy. Mouse urine samples were prepared by using 200 µL of urine mixed with 200 µL of water and 200 µL of 0.1 M phosphate buffer solution (10 % $^{2}H_2O/H_2O$ vol/vol, with 0.05 % sodium 3-trimethylsilyl-(2,2,3,3-$^{2}H_4$)-1-propionate for chemical shift reference at δ-0.0) in 96-well plates for high-throughput flow-injection NMR acquisition. Standard ^{1}H NMR spectra were measured on a spectrometer (Bruker, Rheinstetten, Germany) operating at 600.22 MHz ^{1}H frequency, as described previously described (Dumas et al., 2006). Structural assignment was achieved using ^{1}H-^{13}C Heteronuclear Single Quantum Coherence (HSQC) and ^{1}H-^{1}H Correlation Spectroscopy (COSY) NMR spectroscopy, as well as in-house and publicly available standard assignment databases.

Univariate testing. Univariate General Linear Model (GLM) was performed for phenotype analyses using the SPSS statistical package (version 12.0). To assess differences between mouse groups, Fisher's LSD and Tamhane's T2 post hoc tests were used according to Levene's test for equality of variance. The test for homogeneity of variance was used to determine whether groups had significantly different measures of variability for various parameters.

Multivariate Statistical Analysis of ^{1}H NMR metabolic profiles. The ^{1}H NMR spectra were phased, calibrated and baseline-corrected before being imported into Matlab (R2012b, Mathworks, Natick, MA) at high resolution. The regions δ-6.0–5.5 and δ-5.0–4.5 were removed to eliminate baseline effects of imperfect water signal pre-saturation. The dataset was pre-normalized using row-profile normalization to maintain the internal correlation structure of the dataset. The dataset was then further aligned using RSPA (Veselkov et al., 2009), objective peak identification was performed using statistical recoupling of variables (SRV) (Blaise et al., 2009). Finally, variance-stabilizing logarithmic transform of the SRV clusters (Veselkov et al., 2011) and probabilistic quotient normalization (Dieterle et al., 2006) were used in order to optimize predictive modeling of quantitative physiological variables. Outlier detection was achieved by Principal Component Analysis (PCA). Predictive models were built using Orthogonal Partial Least Squares Discriminant Analysis (O-PLS-DA) (Cloarec et al., 2005), using 7-fold cross-validation (Fonville et al., 2010). Optimal multivariate models were further validated by resampling 10,000 times under the null hypothesis (i.e. generating models with a randomly permuted ***Y*** not related to the disease outcome). The Q^2_{Yhat} goodness-of-prediction statistics of the target model was tested for membership to the population of 10,000 random Q^2_{Yhat} values from the null models and an empirical p-value was derived (Blaise et al., 2007). The metabolic biomarkers predictive of disease outcome were further investigated using analysis of variance (AOV) and *p*-values were adjusted using a subsequent Benjamini and Hochberg multiple testing correction.

Statistical analysis of EPD transcriptome. Quality control checks were performed to confirm the integrity of the microarray data using simpleaffy (http://bioinformatics.picr.man.ac.uk/simpleaffy/). Microarray data were analyzed using R and the BioConductor packages LIMMA (Linear Models for Microarray Data) (Smyth, 2005) and affy (Gautier et al., 2004). Data were background-corrected using rma and normalized using loess. Annotations of genes associated with probes on the microarrays were updated using information from the library mouse4302.db (updated on 12 August 2013). Log_2 scale expression data for 16 animals ($n = 4$ in each group of animals: *L-IGT*, *Mid*, *LNG* and *Ob-IGT*) were correlated against TMAO concentration data using Spearman (corrected for ties). Permutation testing (1,000 replications) was performed for each correlation test to confirm the validity of the significant ($p<0.05$) correlations. Occurrence of significantly correlated genes in

various KEGG pathways was determined. The significance of the over-representation of gene ontology (GO) terms in the list of significantly correlated genes was determined using BiNGO (Maere et al., 2005), returning Cytoscape graphs. An over-representation analysis was done with TMAO-correlated genes and the GO category 'Biological Process'. Only significant (*P*<0.05, after correction for multiple testing using the Benjamini–Hochberg procedure) nodes containing TMAO-correlated genes were retained (*Table S4D*). No annotations were retained for the following entries: 1700011B04RIK, S100A3, D10ERTD447E, KLRA17, 5330422M15RIK, D2ERTD105E, AI854703, 4930505O19RIK, CD52, DNAJB12, CD53, TPD52, 1810013D10RIK, A130094D17RIK, 6430537K16RIK, DXERTD223E, B130034C11RIK, BC065403, FYB, E130119H09RIK, 3100002H20RIK, AU014972, PSMD7, LAIR1, 4931402G19RIK, ANKRA2, 1700058M13RIK, AI463170, 2310045N14RIK, 4930413F20RIK, 5430416O09RIK, 5330423I11RIK, 5830468K08RIK, TNFRSF22, GM15472, GM11827, C77673, 4921517O11RIK, D18ERTD232E, KEG1, TPM3, 5230401M06RIK, 6030458E02RIK, COL9A2, PLEKHO1, 4831407H17RIK, 2810013P06RIK, TCIRG1, MS4A4B, C80993, 4933406K04RIK, 4933433F19RIK, GM20081, GM20083, 7630403G23RIK, GM19835, C030013C21RIK, C030014L02, 8030447M02RIK, D830026I12RIK, TMEM43, GM19544.

Abbreviations: BW_5wk, body weight at 5 weeks (baseline, ie before diet switch); BW_2m, body weight at 2 months (after 3 weeks of HFD); BMI_2m, body mass index at 2 months; FG_2m, fasting glycemia at 2 months; T15_2m, glycemia 15 minutes after glucose injection during the IP-GTT at 2 months; T30_2m, glycemia 30 minutes after glucose injection during the IP-GTT at 2 months; T75_2m, glycemia 75 minutes after glucose injection during the IP-GTT at 2 months; CG_2m, cumulative glycemia during IP-GTT at 2 months; dG_2m, cumulative glycemia over baseline during IP-GTT at 2 months; K15to30_2m, glucose clearance parameter between 15 and 30 minutes during IP-GTT at 2 months; FI_2m, fasting insulinemia at 2 months; I15_2m, plasma insulin 15 minutes after glucose injection during IP-GTT at 2 months; I30_2m, plasma insulin 30 minutes after glucose injection during IP-GTT at 2 months; I75_2m, plasma insulin 75 minutes after glucose injection during IP-GTT at 2 months; CI_2m, cumulative insulinemia (AUC) during IP-GTT at 2 months; IcumG_2m, ratio between cumulative insulinemia and cumulative glycemia during IP-GTT at 2 months; dI_2m, cumulative insulinemia over baseline during IP-GTT at 2 months; K15to75ins_2m, insulin clearance rate during the IP-GTT at 2 months; EPD_wt, epididymal fat pad weight at 2 months; RFP_wt, retroperitoneal fat pad weight at 2 months; BAT_wt, brown adipose tissue weight at 2 months; heart_wt: heart weight at 2 months; EPD_ratio, epididimal fat pad weight normalized to body weight at 2 months; RFP_ratio, retroperitoneal fat pad weight normalized to body weight at 2 months; BAT_ratio, brown adipose tissue weight normalized to body weight at 2 months; heart_ratio, heart weight normalized to body weight at 2 months; food.calc_2m, food consumption calculated at 2 months; ent%.op_2m, number of entries in the open arm at 2 months; t(c) _2m, time to enter open arm at 2 months; dig.energy_2m,

digestible energy calculated at 2 months; rearings.OF_2m, number of rearings in open field test at 2 months; %wtgain5to8w: body weight gain during 3 weeks of HFD from baseline at 5 weeks to 8 weeks (2 months); BCAAs, branched chain amino acids; AKIV, alpha-keto-isovalerate; BAIB, beta-aminoisobutyrate; AKG, 2-oxogutarate; DMG, *N*-*N*-dimethylglycine.

Supplemental References

Blaise, B.J., Giacomotto, J., Elena, B., Dumas, M.-E., Toulhoat, P., Ségalat, L., Emsley, L., 2007. Metabotyping of Caenorhabditis elegans reveals latent phenotypes. Proc. Natl. Acad. Sci. U.S.A. 104, 19808–19812.

Blaise, B.J., Shintu, L., Elena, B., Emsley, L., Dumas, M.-E., Toulhoat, P., 2009. Statistical recoupling prior to significance testing in nuclear magnetic resonance based metabonomics. Anal Chem 81, 6242–6251.

Cani, P.D., Amar, J., Iglesias, M.A., Poggi, M., Knauf, C., Bastelica, D., Neyrinck, A.M., Fava, F., Tuohy, K.M., Chabo, C., Waget, A., Delmée, E., Cousin, B., Sulpice, T., Chamontin, B., Ferrières, J., Tanti, J.-F., Gibson, G.R., Casteilla, L., Delzenne, N.M., Alessi, M.C., Burcelin, R., 2007. Metabolic endotoxemia initiates obesity and insulin resistance. Diabetes 56, 1761–1772.

Cloarec, O., Dumas, M.E., Trygg, J., Craig, A., Barton, R.H., Lindon, J.C., Nicholson, J.K., Holmes, E., 2005. Evaluation of the orthogonal projection on latent structure model limitations caused by chemical shift variability and improved visualization of biomarker changes in ^{1}H NMR spectroscopic metabonomic studies. Anal Chem 77, 517–526.

Dieterle, F., Ross, A., Schlotterbeck, G., Senn, H., 2006. Probabilistic quotient normalization as robust method to account for dilution of complex biological mixtures. Application in 1H NMR metabonomics. Anal Chem 78, 4281–4290.

Dumas, M.-E., Barton, R.H., Toye, A., Cloarec, O., Blancher, C., Rothwell, A., Fearnside, J., Tatoud, R., Blanc, V., Lindon, J.C., Mitchell, S.C., Holmes, E., McCarthy, M.I., Scott, J., Gauguier, D., Nicholson, J.K., 2006. Metabolic profiling reveals a contribution of gut microbiota to fatty liver phenotype in insulin-resistant mice. Proc. Natl. Acad. Sci. U.S.A. 103, 12511–12516.

Fearnside, J.F., Dumas, M.-E., Rothwell, A.R., Wilder, S.P., Cloarec, O., Toye, A., Blancher, C., Holmes, E., Tatoud, R., Barton, R.H., Scott, J., Nicholson, J.K., Gauguier, D., 2008. Phylometabonomic patterns of adaptation to high fat diet feeding in inbred mice. PLoS ONE 3, e1668.

Fonville, J.M., Richards, S.E., Barton, R.H., Boulange, C.L., Ebbels, T.M.D., Nicholson, J.K., Holmes, E., Dumas, M.-E., 2010. The evolution of partial least squares models and related chemometric approaches in metabonomics and metabolic phenotyping. J Chemometr 24, 636–649.

Gautier, L., Cope, L., Bolstad, B.M., Irizarry, R.A., 2004. affy--analysis of Affymetrix GeneChip data at the probe level. Bioinformatics 20, 307–315.

Maere, S., Heymans, K., Kuiper, M., 2005. BiNGO: a Cytoscape plugin to assess overrepresentation of gene ontology categories in biological networks. Bioinformatics 21, 3448–3449.

Smith, P.J., Wise, L.S., Berkowitz, R., Wan, C., Rubin, C.S., 1988. Insulin-like growth factor-I is an essential regulator of the differentiation of 3T3-L1 adipocytes. J. Biol. Chem. 263, 9402–9408.

Smyth, G.K., 2005. limma: Linear Models for Microarray Data, in: … And Computational Biology Solutions Using R and …, Statistics for Biology and Health. Springer-Verlag, New York, pp. 397–420.

Solberg, L.C., Valdar, W., Gauguier, D., Nunez, G., Taylor, A., Burnett, S., Arboledas-Hita, C., Hernandez-Pliego, P., Davidson, S., Burns, P., Bhattacharya, S., Hough, T., Higgs, D., Klenerman, P., Cookson, W.O., Zhang, Y., Deacon, R.M., Rawlins, J.N.P., Mott, R., Flint, J., 2006. A protocol for high-throughput phenotyping, suitable for quantitative trait

analysis in mice. Mamm. Genome 17, 129–146.

Toye, A.A., Dumas, M.E., Blancher, C., Rothwell, A.R., Fearnside, J.F., Wilder, S.P., Bihoreau, M.T., Cloarec, O., Azzouzi, I., Young, S., Barton, R.H., Holmes, E., McCarthy, M.I., Tatoud, R., Nicholson, J.K., Scott, J., Gauguier, D., 2007. Subtle metabolic and liver gene transcriptional changes underlie diet-induced fatty liver susceptibility in insulin-resistant mice. Diabetologia 50, 1867–1879.

Valdar, W., Solberg, L.C., Gauguier, D., Cookson, W.O., Rawlins, J.N.P., Mott, R., Flint, J., 2006. Genetic and environmental effects on complex traits in mice. Genetics 174, 959–984.

Veselkov, K.A., Lindon, J.C., Ebbels, T.M.D., Crockford, D., Volynkin, V.V., Holmes, E., Davies, D.B., Nicholson, J.K., 2009. Recursive segment-wise peak alignment of biological (1)h NMR spectra for improved metabolic biomarker recovery. Anal Chem 81, 56–66.

Veselkov, K.A., Vingara, L.K., Masson, P., Robinette, S.L., Want, E., Li, J.V., Barton, R.H., Boursier-Neyret, C., Walther, B., Ebbels, T.M., Pelczer, I., Holmes, E., Lindon, J.C., Nicholson, J.K., 2011. Optimized preprocessing of ultra-performance liquid chromatography/mass spectrometry urinary metabolic profiles for improved information recovery. Anal Chem 83, 5864–5872.

Wang, F.-M., Chen, Y.-J., Ouyang, H.-J., 2011. Regulation of unfolded protein response modulator XBP1s by acetylation and deacetylation. Biochem. J. 433, 245–252.

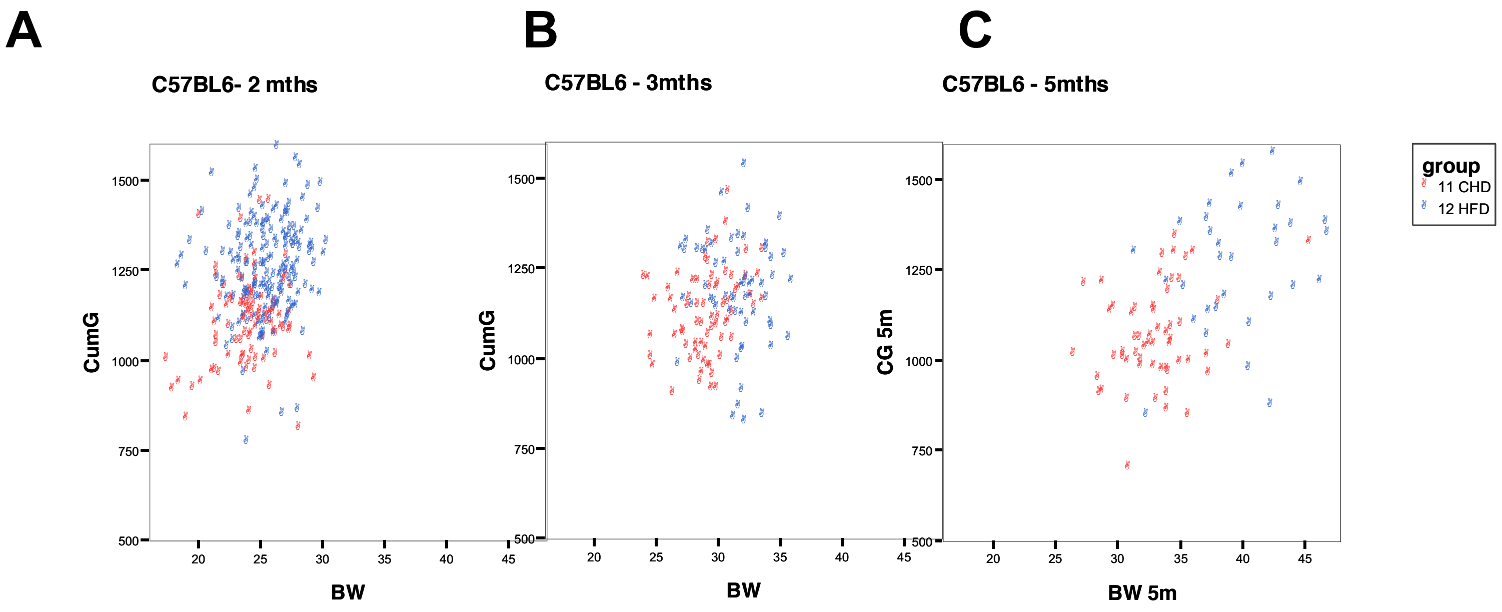

D

	2 months (3 week HFD)		3 months (8 week HFD)		5 months (15 week HFD)	
	**SD(BW)**	**SD(CumG)**	**SD(BW)**	**SD(CumG)**	**SD(BW)**	**SD(CumG)**
CHD	2.21	120.61	2.18	116.61	3.14	132.77
HFD	2.23	143.61	2.68	149.77	3.47	177.45

Figure S1. Preliminary assessment of phenotypic variability in C57BL/6J mice. Two populations of isogenic C57BL/6J fed either CHD (n=145) or HFD (n=193).

A Biplot showing rapid segregation of physiological phenotypes for diabetes (cumulative glycemia, CumG) and obesity (BW) after 3, 7 and 15 weeks.

B Standard deviations associated with the BW vs. CumG scatter plots during the onset of diabetes and obesity.

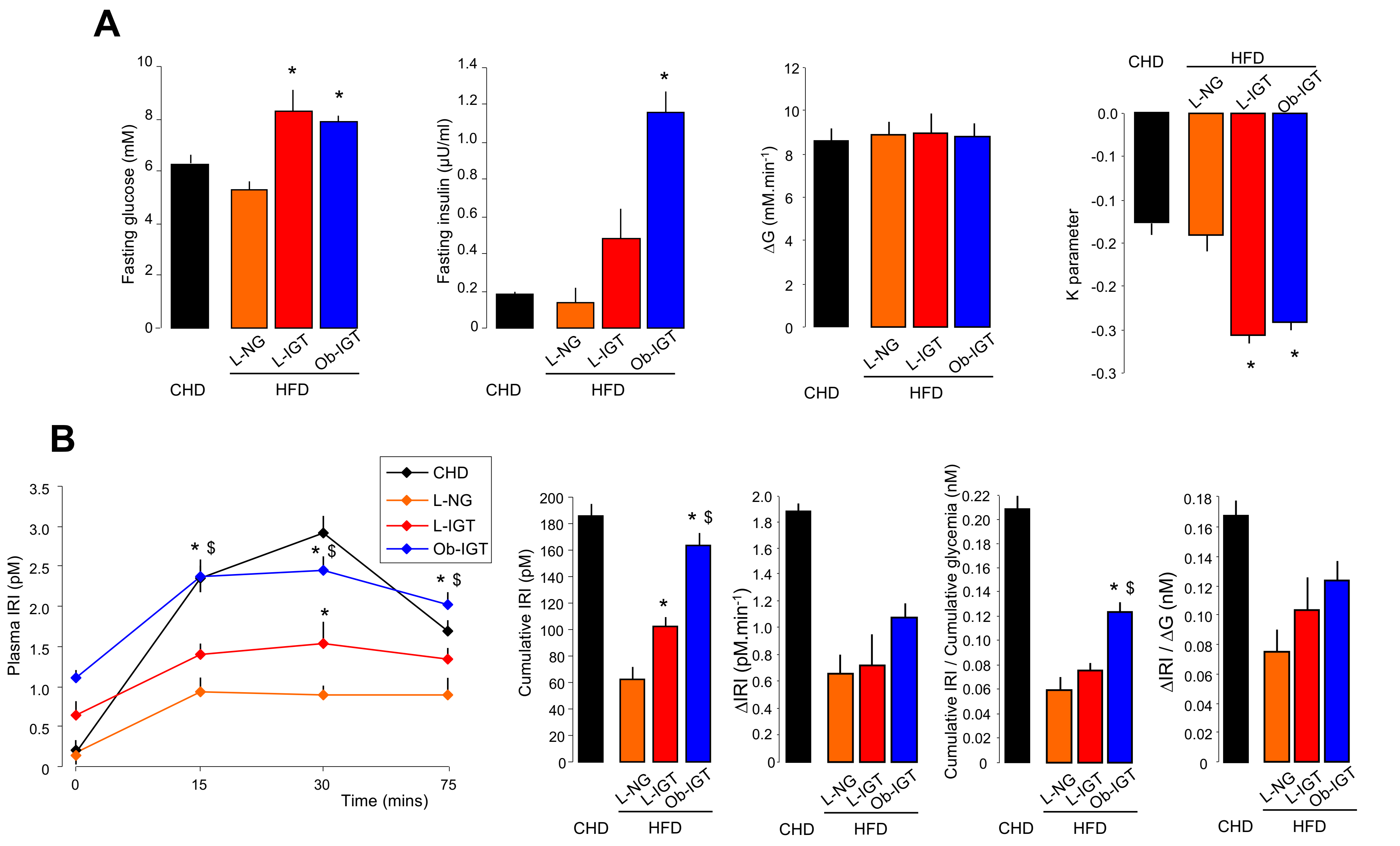

Figure S2. Phenotypic features associated with impaired glucose tolerance and insulin secretion in extreme responders to high fat diet feeding, related to Fig. 1.

S2A Effects of short-term (3 weeks) high fat diet feeding on glucose homeostasis in C57BL/6J mice.

S2B Glucose-stimulated insulin secretion. Age matched controls were fed a standard carbohydrate diet (CHD) (n=50). Three fat-fed groups (n=6) were classified as lean normoglycemic (LNG), lean glucose intolerant (L-IGT), or obese glucose intolerant (Ob-IGT). Data are presented as means ± SE *$p<0.05$, **$p<0.01$, ***$p<0.001$.

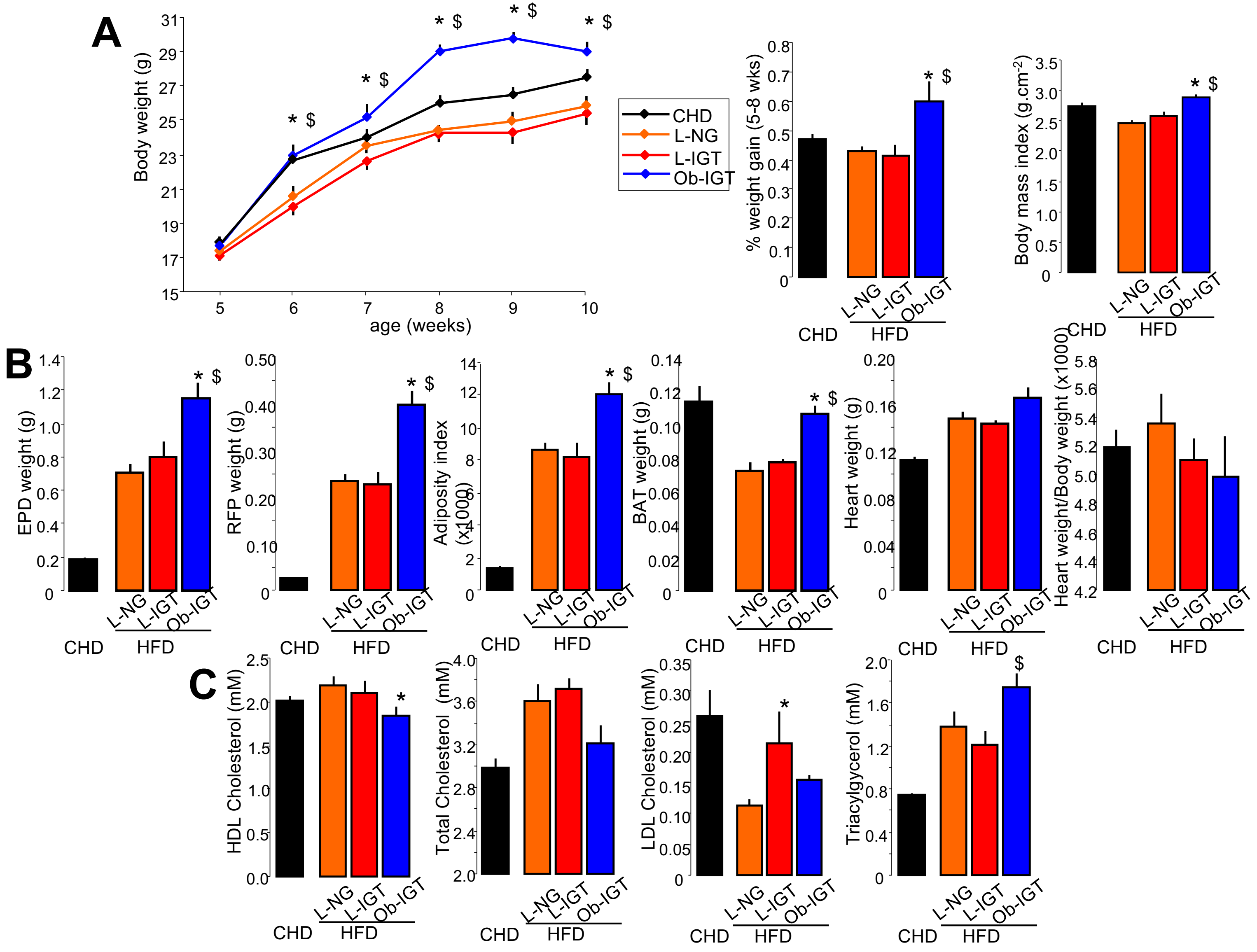

Figure S3. Phenotypic features associated with body weight, organ weight and lipid metabolism in extreme responders to high fat diet feeding, related to Fig. 1.

S3A Effects of high fat diet feeding on the evolution of BW

S3B Effects of high fat diet feeding on the evolution of organ weight and adiposity indices

S3C Effects of high fat diet feeding on the evolution of cholesterol metabolism in C57BL/6J mice.

Age matched controls were fed a standard carbohydrate diet (CHD, n=50). Three fat-fed groups (n=6) were classified as lean normoglycemic (LNG), lean glucose intolerant (L-IGT), or obese glucose intolerant (Ob-IGT). Data are presented as means±SE *$p<0.05$, **$p<0.01$, ***$p<0.001$.

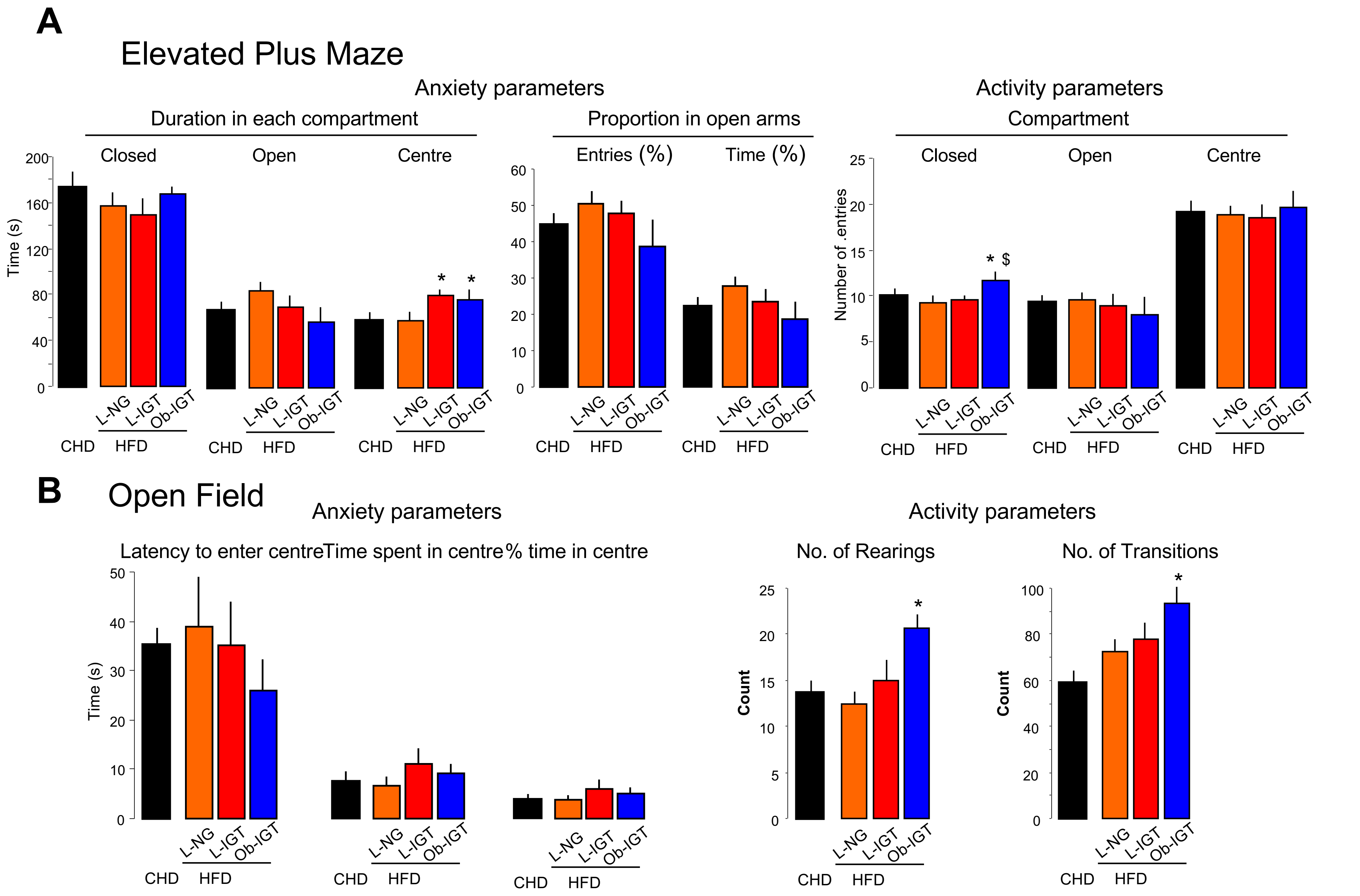

Figure S4. Phenotypic features associated with anxiety and activity in extreme responders to high fat diet feeding, related to Fig. 1.

S4A Elevated plus maze (EPM) activity and anxiety test.

S4B Open Field (OF) activity and anxiety test.

Effects of short-term (3 weeks) high fat diet feeding on anxiety and activity phenotypes determined through elevated plus maze and OF procedures in C57BL6 mice.
