# Supplementary material for: Microbial-Host Co-metabolites Are Prodromal Markers Predicting Phenotypic Heterogeneity in Behavior, Obesity, and Impaired Glucose Tolerance
Source: Cell Rep. 2017 Jul 5;20(1):136–48. doi: 10.1016/j.celrep.2017.06.039 (PMC5507771; doi:10.1016/j.celrep.2017.06.039)
Supplement: Table S1. Metabolite Assignment by 1H-NMR Spectroscopy, Related to Figure 3 [file mmc2.docx]

**Table S1.** Metabolite assignment by ^1^H NMR spectroscopy.

| Compound | Assignment | δ ^1^H | Multiplicity | Confirmation |
| --- | --- | --- | --- | --- |
| acetate | βCH_3_ | 1.92 | s | 1D, COSY, HSQC |
| alanine | βCH_3_ | 1.47 | d | 1D, COSY, HSQC |
| allantoin | CH | 5.38 | s | 1D, COSY, HSQC |
| choline | N-(CH_3_)_3_ | 3.22 | s | 1D, COSY, HSQC |
|  | βCH_2_ | 3.53 | dd | 1D, COSY, HSQC |
|  | αCH_2_ | 4.03 | t | 1D, COSY, HSQC |
| citrate | 1/2γCH | 2.54 | AB | 1D, COSY, HSQC |
|  | 1/2γCH | 2.68 | d | 1D, COSY, HSQC |
| creatine | N-CH_3_ | 3.92 | s | 1D, COSY, HSQC |
|  | N-CH_2_ | 3.03 | s | 1D, COSY, HSQC |
| creatinine | N-CH_3_ | 3.03 | s | 1D, COSY, HSQC |
|  | N-CH_2_ | 4.05 | s | 1D, COSY, HSQC |
| dimethylamine (DMA) | CH_3_ | 2.5 | s | 1D, COSY, HSQC |
| dimethylglycine (DMG) | CH_2_ | 3.72 | s | 1D, COSY, HSQC |
| ethanolamine | CH_2_NH_2_ | 3.14 | t | 1D, COSY, HSQC |
| formate | HCOOH | 8.46 | s | 1D, COSY, HSQC |
| fumarate | C1H | 6.51 | s | 1D, COSY, HSQC |
| α-D-glucose | H_1_ | 5.24 | d | 1D, COSY, HSQC |
| β-D-glucose | H_1_ | 4.66 | d | 1D, COSY, HSQC |
| glycine | αCH_2_ | 3.55 | s | 1D, COSY, HSQC |
| hippurate | CH_2_ | 3.96 | d | 1D, COSY, HSQC |
|  | *m*CH | 7.54 | t | 1D, COSY, HSQC |
|  | *p*CH | 7.63 | t | 1D, COSY, HSQC |
|  | *o*CH | 7.82 | d | 1D, COSY, HSQC |
| hexanoyl-glycine | CH_3_ | 0.9 | t | 1D, COSY, HSQC |
|  | (CH_2_) | 1.6 | m | 1D, COSY, HSQC |
| isobutyrate | CH_3_ | 1.21 | d | 1D, COSY, HSQC |
| isovaleryl-glycine | ε CH_3_ | 0.925 | d | 1D, COSY, HSQC |
|  | γCH_2_ | 2.16 | d | 1D, COSY, HSQC |
| 3-methyl-2-oxovalerate | CH_3_ | 1.1 | d | 1D, COSY, HSQC |
| lactate | CH_3_ | 1.33 | d | 1D, COSY, HSQC |
|  | αCH | 4.12 | q | 1D, COSY, HSQC |
| leucine | (CH_3_)_2_ | 0.94 | t | 1D, COSY, HSQC |
| mono-methylamine | N-CH_3_ | 2.92 | s | 1D, COSY, HSQC |
| 2-oxoglutarate | CH | 2.43 | t | 1D, COSY, HSQC |
| 2-oxoisovalerate | CH_3_ | 1.11 | d | 1D, COSY, HSQC |
| 4-cresol-sulfate | CH_3_ | 7.05 | d | 1D, COSY, HSQC |
|  | C_3_H & C_5_H | 7.21 | t | 1D, COSY, HSQC |
| phenylacetylglycine (PAG) | CH_2_ | 3.68 | s | 1D, COSY, HSQC |
|  | CH_2_ | 3.75 | d | 1D, COSY, HSQC |
|  | C_3_H & C_5_H | 7.43 | m | 1D, COSY, HSQC |
| putrescine | (CH_3_)_2_ | 1.75 | m | 1D, COSY, HSQC |
| pyruvate | CH_3_ | 2.46 | s | 1D, COSY, HSQC |
| raffinose | CH_3_ (fructose) | 4.23 | d | 1D, COSY, HSQC |
|  | CH_2_ (galactose) | 5 | d | 1D, COSY, HSQC |
|  | CH (sucrose) | 5.4 | d | 1D, COSY, HSQC |
| succinate | CH_2_-COOH | 2.39 | s | 1D, COSY, HSQC |
| taurine | N-CH_2_ | 3.26 | t | 1D, COSY, HSQC |
|  | S-CH_2_ | 3.34 | t | 1D, COSY, HSQC |
| trimethylamine (TMA) | N-(CH_3_)_3_ | 2.88 | s | 1D, COSY, HSQC |
| trimethylamine-*N*-oxide (TMAO) | N-(CH_3_)_3_ | 3.25 | s | 1D, COSY, HSQC |
| tyrosine | C_3_H & C_5_H | 6.91 | d | 1D, COSY, HSQC |
|  | C_2_H & C_6_H | 7.19 | d | 1D, COSY, HSQC |
| valine | γCH_3_ | 0.98 | d | 1D, COSY, HSQC |
|  | βCH | 2.27 | m | 1D, COSY, HSQC |

^1^H chemical shift data for metabolites of urine samples, showing compound name, chemical group, chemical shift in parts per million (ppm), multiplicity and types of spectra. Abbreviations: COSY, ^1^H-^1^H Correlation spectroscopy; HSQC, ^1^H-^13^C Heteronuclear Single Quantum Coherence spectroscopy.
